# Immunogenic dynamics and SARS-CoV-2 variant neutralisation of the heterologous ChAdOx1-S/BNT162b2 vaccination: Secondary analysis of the randomised CombiVacS study

**DOI:** 10.1016/j.eclinm.2022.101529

**Published:** 2022-07-01

**Authors:** Javier García-Pérez, María González-Pérez, María Castillo de la Osa, Alberto M. Borobia, Luis Castaño, María Jesús Bertrán, Magdalena Campins, Antonio Portolés, David Lora, Mercedes Bermejo, Patricia Conde, Lourdes Hernández-Gutierrez, Antonio Carcas, Eunate Arana-Arri, Marta Tortajada, Inmaculada Fuentes, Ana Ascaso, María Teresa García-Morales, Humberto Erick de la Torre-Tarazona, José-Ramón Arribas, Natale Imaz-Ayo, Eugènia Mellado-Pau, Antonia Agustí, Carla Pérez-Ingidua, Agustín Gómez de la Cámara, Jordi Ochando, Cristobal Belda-Iniesta, Jesús Frías, José Alcamí, Mayte Pérez-Olmeda, CombiVacS study Group

**Affiliations:** a Unidad de Inmunopatología del SIDA, Centro Nacional de Microbiología, Instituto de Salud Carlos III (ISCIII), Madrid, Spain; b Centro de Investigación Biomédica en Red de Enfermedades Infecciosas (CIBERINFEC), Instituto de Salud Carlos III (ISCIII), Madrid, Spain; c Laboratorio de Referencia en Inmunología, Centro Nacional de Microbiología, Instituto de Salud Carlos III (ISCIII), Madrid, Spain; d Laboratorio de Serología, Centro Nacional de Microbiología, Instituto de Salud Carlos III (ISCIII), Madrid, Spain; e Servicio de Farmacología Clínica, Departamento de Farmacología y Terapéutica, Facultad de Medicina, Hospital Universitario La Paz, IdiPAZ, Universidad Autónoma de Madrid, Madrid, Spain; f Biocruces Bizkaia, Hospital Universitario Cruces, CIBERDEM, CIBERER, Endo-ERN, UPV-EHU, Barakaldo, Spain; g Servicio de Medicina Preventiva y Epidemiologia, Hospital Clínic de Barcelona, Barcelona, Spain; h Servicio de Medicina Preventiva y Epidemiología, Hospital Universitari Vall d'Hebron, Universitat Autònoma de Barcelona, Barcelona, Spain; i Servicio de Farmacología Clínica, Hospital Clínico San Carlos, IdISSC, Madrid, Spain; j Departamento de Farmacología y Toxicología, Facultad de Medicina, Universidad Complutense de Madrid (UCM), Madrid, Spain; k Instituto de Investigación Sanitaria Hospital 12 de octubre (imas12), Facultad de Medicina, Universidad Complutense de Madrid (UCM); l Centro de Investigación Biomédica en Red de Epidemiología y Salud Pública (CIBERESP), Instituto de Salud Carlos III (ISCIII), Madrid, Spain; m Servicio de Prevención de Riesgos Laborales, Salud Laboral, Hospital Clínic de Barcelona, Barcelona, Spain; n Unidad de Soporte a la Investigación Clínica, Vall d'Hebron Institut de Recerca, Barcelona, Spain; o Servicio de Medicina Interna, Departamento de Medicina, Facultad de Medicina, Hospital Universitario La Paz, IdiPAZ, Universidad Autónoma de Madrid, Madrid, Spain; p Servicio de Farmacología Clínica, Hospital Universitari Vall d'Hebron, Barcelona, Spain; q Departament de Farmacologia, Terapèutica i Toxicologia, Universitat Autònoma de Barcelona, Bellaterra, Barcelona, Spain; r Facultad de Medicina, Universidad Complutense de Madrid (UCM), Madrid, Spain; s Spanish Clinical Research Network – SCReN – ISCIII, Madrid, Spain; t Directorate Instituto de Salud Carlos III, Madrid, Spain; u Facultad de Estudios Estadísticos, Universidad Complutense de Madrid (UCM), Madrid, Spain

**Keywords:** SARS-CoV-2, Heterologous vaccination, Neutralisation, Variants, Antibodies

## Abstract

**Background:**

The CombiVacS study was designed to assess immunogenicity and reactogenicity of the heterologous ChAdOx1-S/BNT162b2 combination, and 14-day results showed a strong immune response. The present secondary analysis addresses the evolution of humoral and cellular response up to day 180.

**Methods:**

Between April 24 and 30, 2021, 676 adults primed with ChAdOx1-S were enrolled in five hospitals in Spain, and randomised to receive BNT162b2 as second dose (interventional group [IG]) or no vaccine (control group [CG]). Individuals from CG received BNT162b2 as second dose and also on day 28, as planned based on favourable results on day 14. Humoral immunogenicity, measured by immunoassay for SARS-CoV-2 receptor binding domain (RBD), antibody functionality using pseudovirus neutralisation assays for the reference (G614), Alpha, Beta, Delta, and Omicron variants, as well as cellular immune response using interferon-γ and IL-2 immunoassays were assessed at day 28 after BNT162b2 in both groups, at day 90 (planned only in the interventional group) and at day 180 (laboratory data cut-off on Nov 19, 2021). This study was registered with EudraCT (2021-001978-37) and ClinicalTrials.gov (NCT04860739).

**Findings:**

In this secondary analysis, 664 individuals (441 from IG and 223 from CG) were included. At day 28 post vaccine, geometric mean titres (GMT) of RBD antibodies were 5616·91 BAU/mL (95% CI 5296·49–5956·71) in the IG and 7298·22 BAU/mL (6739·41–7903·37) in the CG (*p* < 0·0001). RBD antibodies titres decreased at day 180 (1142·0 BAU/mL [1048·69–1243·62] and 1836·4 BAU/mL [1621·62–2079·62] in the IG and CG, respectively; *p* < 0·0001). Neutralising antibodies also waned from day 28 to day 180 in both the IG (1429·01 [1220·37–1673·33] and 198·72 [161·54–244·47], respectively) and the CG (1503·28 [1210·71–1866·54] and 295·57 [209·84–416·33], respectively). The lowest variant-specific response was observed against Omicron-and Beta variants, with low proportion of individuals exhibiting specific neutralising antibody titres (NT50) >1:100 at day 180 (19% and 22%, respectively).

**Interpretation:**

Titres of RBD antibodies decay over time, similar to homologous regimes. Our findings suggested that delaying administration of the second dose did not have a detrimental effect after vaccination and may have improved the response obtained. Lower neutralisation was observed against Omicron and Beta variants at day 180.

**Funding:**

Funded by Instituto de Salud Carlos III (ISCIII).

**Research in context**

**Evidence before this study**

We did not perform a systematic search of the literature because of the rapidly developing situation. In summer 2021, the Delta variant of SARS-CoV-2 emerged and replaced the circulating Alpha strain. In November 2021 the Omicron variant was first described, and became predominant by March 2022 worldwide. In parallel, in summer 2021 different works pointed to a decrease in vaccine protection after six months of immunisation, in particular against new variants, due to waning of neutralising antibody activity. Together, these observations open the debate on a third dose booster that was adopted in many countries over the final months of 2021.

**Added value of this study**

The present results provide additional evidence on late immunogenicity – up to 6 months – of the heterologous ChAdOx1-S/BNT162b2 vaccination regime. Further, SARS-CoV-2 variant-specific elicited immunity is also reported here.

**Implications of all the available evidence**

Antibody titres decay over time, but delay in second dose administration has no deleterious effect on the immune response; on the contrary it resulted in better humoral responses. Functionally, all individuals exhibited neutralising titres >1:100 against G614 reference strain at day 28 after vaccine and a relevant proportion (76%) did so at day 180. However, variant-specific neutralisation was variable, with the lowest activity observed at day 180 against Omicron variant followed by Beta and Delta (19%, 22% and 56% with NT50>1:100, respectively). These findings support the use of heterologous regimes, which is consistent with that arising from homologous schemes, and a third dose strategy in patients previously immunised with a combination of adenovirus- and mRNA-based vaccines.

Alt-text: Unlabelled box

## Introduction

Early after the SARS-CoV-2 outbreak started – rapidly evolving to a worldwide pandemic – active immunization emerged as the key priority of global healthcare policies. Most scientific efforts have focused in vaccines development, which successfully resulted in three homologous two-dose vaccines and one single-dose vaccine available for use in the European Union between late 2020 and early 2021. Notwithstanding, rare severe thrombotic with thrombocytopenia events related to ChAdOx1-S vaccine (Vaxzevria, AstraZeneca) and shortage in supplies had an impact on European vaccination plans and drove an interest in heterologous regimes. The combination of ChAdOx1-S and the mRNA vaccine BNT162b2 (Comirnaty, BioNTech) has been the first heterologous scheme studied. Results on 14-day^1^ and 28-day^2^ immunogenicity showed that robust humoral and cellular immune responses were elicited. Accordingly, and as planned in the protocol, participants included in the control group were offered to receive BNT162b2 as a second dose.

Concurrently, public health plans to control the pandemic faced a recurrent issue, namely the periodic outbreak of new SARS-CoV-2 variants. To date, numerous variants have been identified, of which Beta (B.1.351), Gamma (P.1), and Delta (B.1.617.2) are currently considered as variants of concern (VoC), the latter beginning in summer 2021 and soon becoming dominant.^3^ More recently a new variant – Omicron (B.1.1.529) – was first described in Botswana and South Africa. Because of its high transmissibility, Omicron is displacing Delta as the dominant variant in most world countries in less than 1 month, representing a new challenge in the control of pandemic.^4^ Not surprisingly, dynamics over time of variant-specific immune response induced by vaccination is a hot matter of research. Several works have reported a deterioration of vaccine-induced antibody response and a waning of protection against infection with either homologous regimes.^5^^,^^6^ By variants, a 3·5-fold to 14-fold reduction of serum neutralisation titres against Beta variant from vaccinated individuals has been reported.^7^, ^8^, ^9^ Further, ChAdOx1-S vaccine results evidenced undetectable neutralisation activity against Beta variant in 60% of vaccinated individuals and decay by a factor of up to 31·5% in the remaining 40%.^10^ Preliminary data from Omicron variant were even more worrying as a 14 to 30-fold reduction in neutralisation susceptibility elicited by immunisation was reported.^11^^,^^12^

Notwithstanding this, the impact of waning neutralising antibodies on clinical efficacy is not clear. Some studies found that protection against hospitalization or death persisted at a robust level,^6^ while others showed that efficacy notably decreased, or even failed, against Beta^10^^,^^13^ and Delta^6^ variants. A consequent question is whether this reduction makes the variant resistant to vaccination. Some results on Beta variant comparing sera from naturally infected and vaccinated individuals have suggested that those vaccinated retain protective levels of humoral immunity,^7^ while others evidenced no efficacy in mild and moderate disease.^10^ It must be mentioned that effectiveness may be influenced by the interval between doses, with longer time associated to an enhanced antibody response^14^ and higher effectiveness, as well as by cellular immunity, given the relevant role of CD4+ and CD8+ responses found in COVID-19 patients and cross-reactivity observed in unexposed individuals.^15^^,^^16^ Also, as suggested for Omicron variant, a decreased virulence could contribute to lower the rates of hospital admissions and death.^17^^,^^18^

In addition, decay of variant-specific antibody titres is shifting focus towards the need of a third dose, especially in those with weakened immune systems such as older adults^19^ and immunosuppressed patients.^20^^,^^21^ Considering the available evidence, the European Medicines Agency (EMA) has issued recommendations for an extra dose with mRNA vaccines and is completing conclusions on booster doses for people with a normal immune system.^22^

Increasing evidence on immunity dynamics aiming to answer these questions derives mostly from homologous vaccines, while heterologous regimes are still less studied. Here we present additional results of the CombiVacS study^1^ addressing a) total and neutralising antibody dynamics of heterologous ChAdOx1-S/BNT162b2 vaccines combination, and b) immune response against different SARS-CoV-2 variants, including Delta and Omicron variants. This analysis also aims to provide valuable data to the debate on extra booster doses.

## Methods

### Study design and participants

Data from the 12-month, phase 2, open-label, randomised, controlled CombiVacS study are included in this secondary analysis. Full descriptions of the methods as well as safety and initial immunogenicity analyses have been previously published in detail.^1^ Full study protocol is provided in Appendix 1 (p 26).

Healthy, or clinically stable, adults from 18 to 59 years old with no history of SARS-CoV-2 infection who had been vaccinated with a single dose of ChAdOx1-S between 8 and 12 weeks before screening were enrolled in the CombiVacS study to evaluate immunogenicity and reactogenicity of a second dose of the mRNA COVID-19 vaccine BNT162b2.

All participants provided written informed consent before enrolment. The trial complies with the principles of the Declaration of Helsinki and Good Clinical Practice. This study was approved by the Spanish Agency of Medicines and Healthcare Products (AEMPS) and by the Ethics Committee at University Hospital La Paz.

### Randomisation and masking

Briefly, participants were randomly assigned (2:1) to receive one intramuscular injection of BNT162b2 (interventional group, IG) or maintain observation (control group, CG). Since the main immunogenicity objective was met, and reactogenicity was acceptable,^1^ participants included in the control group were offered to receive BNT162b2 as a second dose at day 28, as planned in the protocol. A systematic randomisation stratified by study site, gender and age (18-49 years, and 50-59 years) was used. The randomization list was centrally generated with the SAS software for Windows (version 9.4; SAS Institute Inc., Cary, NC, USA), and imported into the secure Research Electronic Data Capture platform (REDCap version 8.7.4; Vanderbilt University, Nashville, TN, USA) used for the study electronic case report form (eCRF).

### Procedures

Study procedures have been described in full previously.^1^ In brief, at randomization clinical assessments were performed and blood samples for safety and immunology collected from all participants. Concurrently, participants in the interventional group were administered 0·3 mL BNT162b2 dose as a single intramuscular injection (day 0), whilst individuals from control group were vaccinated on day 28 of study. Planned follow-up visits for safety and immunologic purposes were scheduled on days 7, 14, 28, 90, 180 and 360. All vaccinated participants were on-site monitored for safety for at least 15 minutes. Safety procedures included both direct report from individuals during the post-vaccine observation period and online report using an electronic diary throughout the study follow-up period.

To the present analysis, the commercial electrochemiluminescence immunoassay (ECLIA) Elecsys® Anti-SARS-CoV-2 S assay (Roche Diagnostics GmbH, Mannheim, Germany) was used to detect antibodies (including IgG) specific to the SARS-CoV-2 spike protein receptor binding domain (RBD-S protein) on the Cobas e411 module (Roche Diagnostics GmbH, Mannheim, Germany),^23^ with a measuring range from 0·4 to 250 U/mL (up to 2,500 U/mL with onboard 1:10 dilution and up on 12,500 with onboard 1:50 dilution). Values higher than 0·8 BAU/mL were considered positive.

Measurement of neutralising antibodies titres in a predefined subset of 198 participants was carried out by preincubation of diluted plasma samples with titrated pseudoviruses (10 ng p24Gag per well) generated by co-transfection of pNL4-3ΔenvRen and an expression vector for the different viral spikes (pcDNA3.1-S-CoV2∆19-G614, -Alpha, -Beta, -Delta, or -Omicron) and added to Vero E6 cells in 96-well plates. Viral infectivity 48 hours post infection was assessed by measuring luciferase activity (Renilla Luciferase Assay, Promega, Madison, WI, USA) using a 96-well plate luminometer LB 960 Centro XS³ (Berthold Technologies, Oak Ridge, TN, USA). The titre of neutralising antibodies was calculated as 50% inhibitory dose (neutralising titre 50, NT50), expressed as reciprocal of four-fold serial dilution of heat-inactivated sera (range 1:32–1:131·072) resulting in a 50% reduction of pseudovirus infection compared to control without serum. Samples below the detection threshold (1:32 serum dilution) were given 1:16 value. Positive and negative controls were included in the assays and non-specific neutralisation was assessed using a related pseudovirus expressing the vesicular stomatitis virus envelope (VSV-G). Cellular immune response was measured in participants from two pre-selected sites by quantification of interferon-γ (IFN-γ) and interleukin-2 (IL-2) present in plasma on overnight stimulation of whole blood cultured with pools of SARS-CoV-2 spike peptides (2 μg/mL) or dimethyl sulfoxide control. Cytokines were quantified using the next-generation enzyme-linked immunosorbent assay (ELISA) tool, Ella (ProteinSimple, San Jose, CA, USA). Full details on the pseudo-virus neutralisation assays and cellular immunity quantification are provided in the Appendix 1 (pp 18-19).

### Outcomes

Outcomes included in the present secondary analysis were humoral immune response to vaccination as per antibodies titres and neutralising antibody titres at days 28, 90 and 180 after the BNT162b2 dose. Of note, in the control group outcomes at day 28 post-vaccine correspond to day 56 of study, and outcomes at day 180 correspond to day 152 post vaccine. In the control group, no outcomes were planned at day 90, according to the protocol. Alpha-, Beta-, Delta-, and Omicron-specific neutralising antibody titres at days 28 and 180 post-dose have been analysed in both study groups. Cellular response defined as inflammatory IFN-γ and IL-2 cytokines production against SARS-CoV-2 spike peptide pools at days 28 and 180 post-BNT162b2 dose were also assessed.

### Statistical analysis

To the present analysis, the immunogenicity population included all the participants who were randomly assigned, completed all applicable visits, and for whom serological samples were available at the baseline visit and on days 28, 56 (only applicable to control group), 90 (only applicable to interventional group) and 180. Laboratory data cut-off was on November 19, 2021. Day-28 variables (i.e. humoral immunogenicity by RBD-specific IgG analysis, neutralising activity of SARS-CoV-2-specific antibodies, and cellular immunity) were analysed in 658, 194 and 114 individuals, respectively. Missing values from later visits were not imputed (Appendix 1 p 17).

Data were presented as geometric mean and 95% confidence interval (CI) or, for categorical variables, number, and percentage, unless otherwise stated. For serological measurements, difference at each time point was evaluated using ratio of geometric means. Since the outcome variable, i.e. antibodies against SARS-CoV-2 spike protein RBD on day 28 post-dose, was restricted by technical limitations, a truncated regression model was used. The model incorporated right censoring values, raw data response with distribution lognormal data, and treatment effect (interventional group versus control group) adjusted by sex, age, and time between vaccine doses. Additionally, reverse cumulative distribution curve (RCDC) was plotted. A stratified analysis by sex, age and interval between vaccine doses was done for the humoral and cellular immunity endpoints. Laboratory parameters with values below detection limit were replaced by a value equal to the lowest limit divided by two. All analyses were carried out using the statistical software SAS, version 9·4. Sample size was calculated for primary efficacy endpoint – i.e. antibody titres at day 14 –, and was also considered appropriate to evaluate most of secondary endpoints (Appendix 1 p 14). Sample size calculation methods as well as independent data monitoring committee procedures have been described previously.^1^

Composition of the independent data monitoring committee is provided in the Appendix 1 (p 23). This study was registered with EudraCT (2021-001978-37) and ClinicalTrials.gov (NCT04860739).

### Role of the funding source

The funder – Institute of Health Carlos III (ISCIII), a public research organization – designed the trial in cooperation with the Spanish Clinical Trials Platform (SCReN), a public network of clinical trial units at the Spanish National Health System funded by the ISCIII. Trial coordination, participant recruitment and data analysis were performed by SCReN. All immunological procedures were performed at ISCIII. All authors reviewed and approved the original draft. All authors had full access to the full data in the study and accept responsibility to submit for publication.

## Results

**Figure 1:**
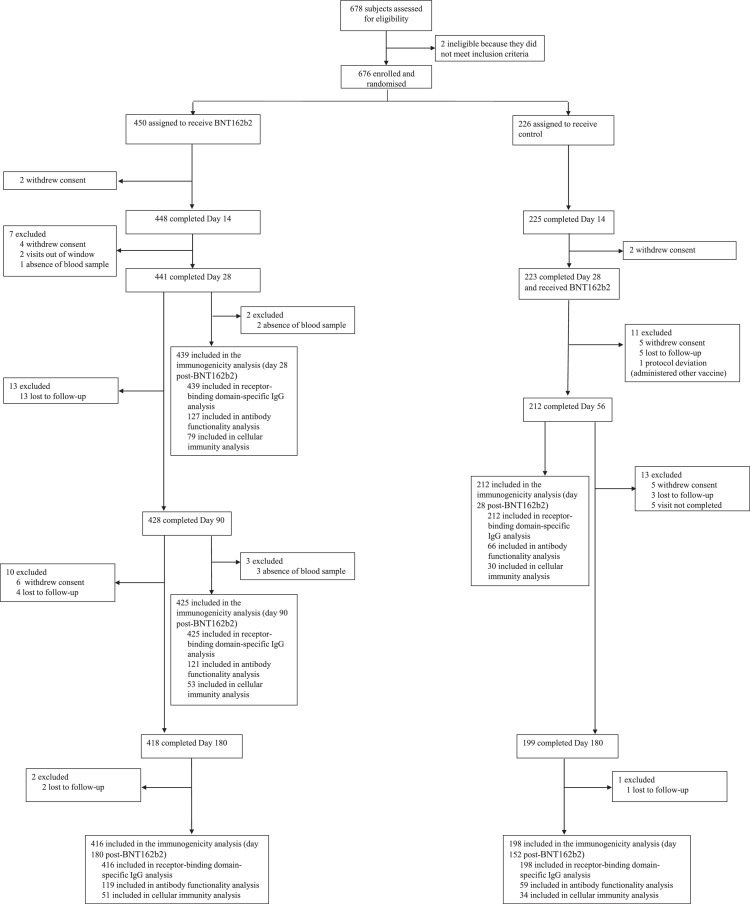
Trial profile.

Of 676 participants enrolled and randomised between April 24^th^ and 30^th^, 2021, 450 individuals were assigned to the interventional group, receiving BNT162b2 as second dose, and 226 were to the control group, maintaining observation. Participant´s flow up to day 14 of study has been fully described previously.^1^ 441 participants from the interventional group completed day 28 of study and 418 completed day 180. 223 individuals from control group received BNT162b2 vaccination at day 28; 212 and 199 of them completed day 56 of study (day 28 post vaccination) and day 180 (day 152 post vaccine), respectively (Figure 1).

Baseline and demographic characteristics of the population are summarised in Table 1. 378 (57%) individuals were women and 289 (43%) were men. 431 (65%) participants were aged 18–49 years, and the mean age of both groups was 44·03 years (SD 8·82). In the control group, mean (SD) interval between ChAdOx1-S and BNT162b2 administration was 89·03 days (5·92). Neither differences between groups were found in demographic characteristics at day 180 (Appendix 1 p 15).

**Table 1:** Baseline characteristics of the population.

	Interventional group (*n* = 450)	Control group (*n* = 226)^d^	Overall (*n* = 676)
**Sex, n (%)**			
Male	193 (43%)	101 (45%)	294 (43%)
Female	257 (57%)	125 (55%)	382 (57%)
**Age (years), mean (SD)**	43·93 (8·88)	44·10 (8·82)	43·98 (8·85)
**Age group, n (%)**			
18-49 years	293/450 (65%)	144/226 (64%)	437/676 (65%)
Male	123/293 (42%)	65/144 (45%)	188/437 (43%)
Female	170/293 (58%)	79/144 (55%)	249/437 (57%)
50-59 years	157/450 (35%)	82/226 (36%)	239/676 (35%)
Male	70/157 (45%)	36/82 (44%)	106/239 (44%)
Female	87/157 (55%)	46/82 (56%)	133/239 (56%)
**Days between vaccines, mean (SD)**	61·16 (5·73)	89·03 (5·92)	70·33 (14·32)

d Based on 223 subjects (3 subjects withdrew consent before being immunized).

Median time in collection of day-28 post- BNT162b2 dose blood sample was similar in both study groups (28 days [interventional] vs. 27 [control]), however variability was higher in individuals from the control group (range 21-38 days [interventional] vs. 16-42 [control]) (Appendix 1 p 2).

**Figure 2:**
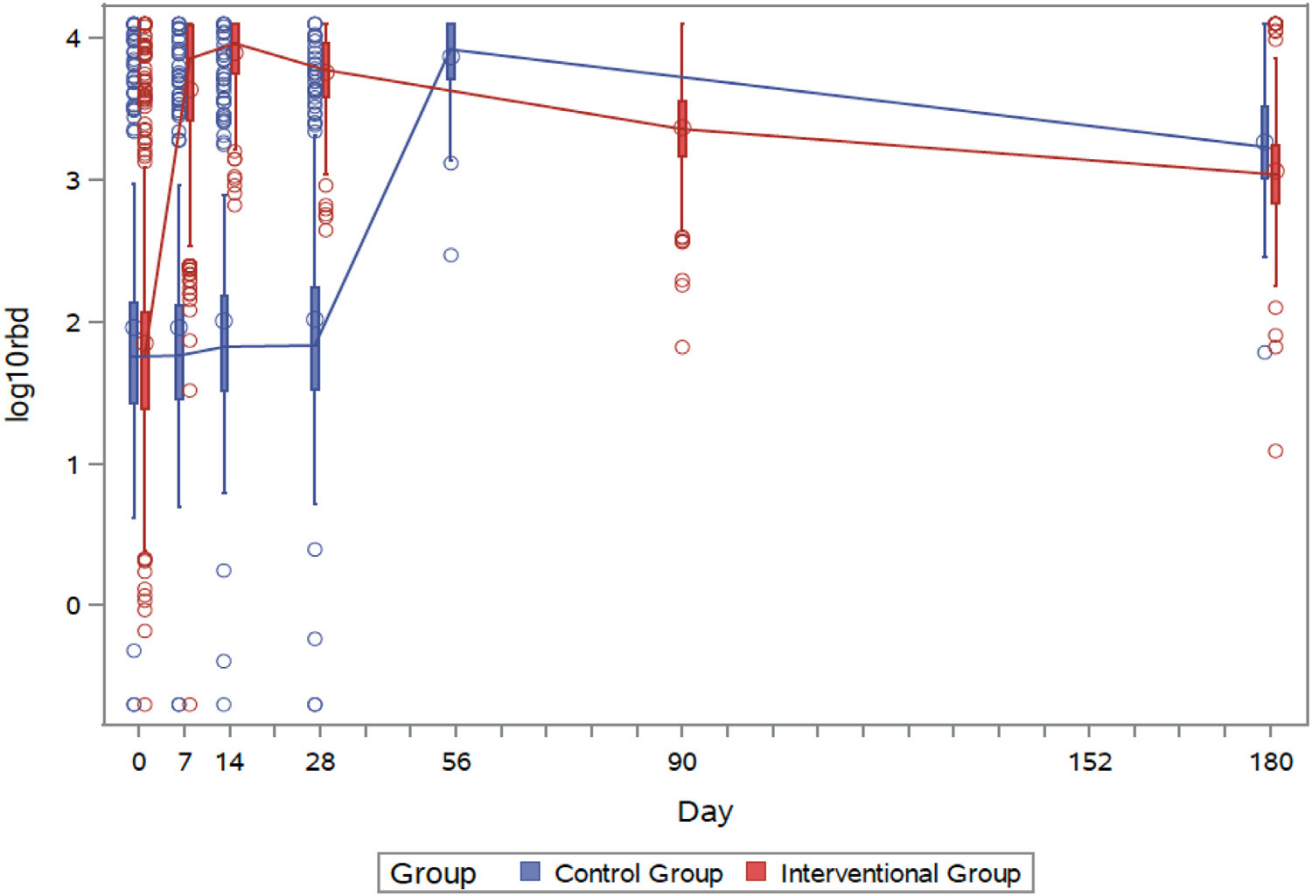
RBD (anti-spike) antibody titres measured in both interventional (red) and control (blue) groups over time. Interventional group was immunised at day 0 and control group at day 28. Accordingly day 180 corresponds to day 152 post vaccine in the control group.

Results on immunogenicity dynamics in both groups show a decay in titres of antibodies specific to the SARS-CoV-2 S-protein RBD over time (Appendix 1 pp 3-4). In the interventional group, geometric mean titres (GMT) of S-RBD antibodies decreased from 7739·21 BAU/mL (95% CI 7371·53–8161·96) at day 14 after BNT162b2 second dose (fully reported earlier)^1^ to 5616·91 BAU/mL (95% CI 5296·49–5956·71) at day 28, 2303·28 BAU/mL (95% CI 2141·66–2477·1) at day 90 and 1142·0 BAU/mL (1048·69–1243·62) at day 180. Of note, waning was slower from third to six month (mean lognormal difference -0·303 [95% CI -0·324–(-)0·283]) than from first to third (-0·389 [95% CI -0·405–(-)0·374]). The regression model for outcome variables to 180 days by treatment (interventional group versus control group) and adjusted by covariables is showed in the Appendix 1 p 16. Interestingly, immunogenic response in the control group was significantly stronger at day 28 after second dose (7298·22 BAU/mL [95%CI 6739·41–7903·37]) than in the interventional group (*p* < 0·0001). Likewise, antibody levels remained higher at day 180 in the control compared to interventional group (1836·4 BAU/mL [95%CI 1621·62–2079·62]; *p* < 0·0001) (Figure 2; Appendix 1 p 3). This effect was also observed in stratified analyses by sex and age (Appendix 1 pp 5–6). Adjusted differences in day-28 and day-180 lognormal RBD values in the interventional vs. control group resulted from the regression model were -0·0881 (95% CI -0·1239–(-)0·0523 [*p* < 0·0001]) and -0·1760 (95% CI -0·2277–(-)0·1242 [*p* < 0·0001]), respectively. In addition, the effect of delayed vaccination in the control group is linear over time, resulting in a difference of S-RBD antibody levels in the test vs. control group of -0·1329 (95% CI -0·1831–(-)0·0826) over the follow-up period.

**Figure 3:**
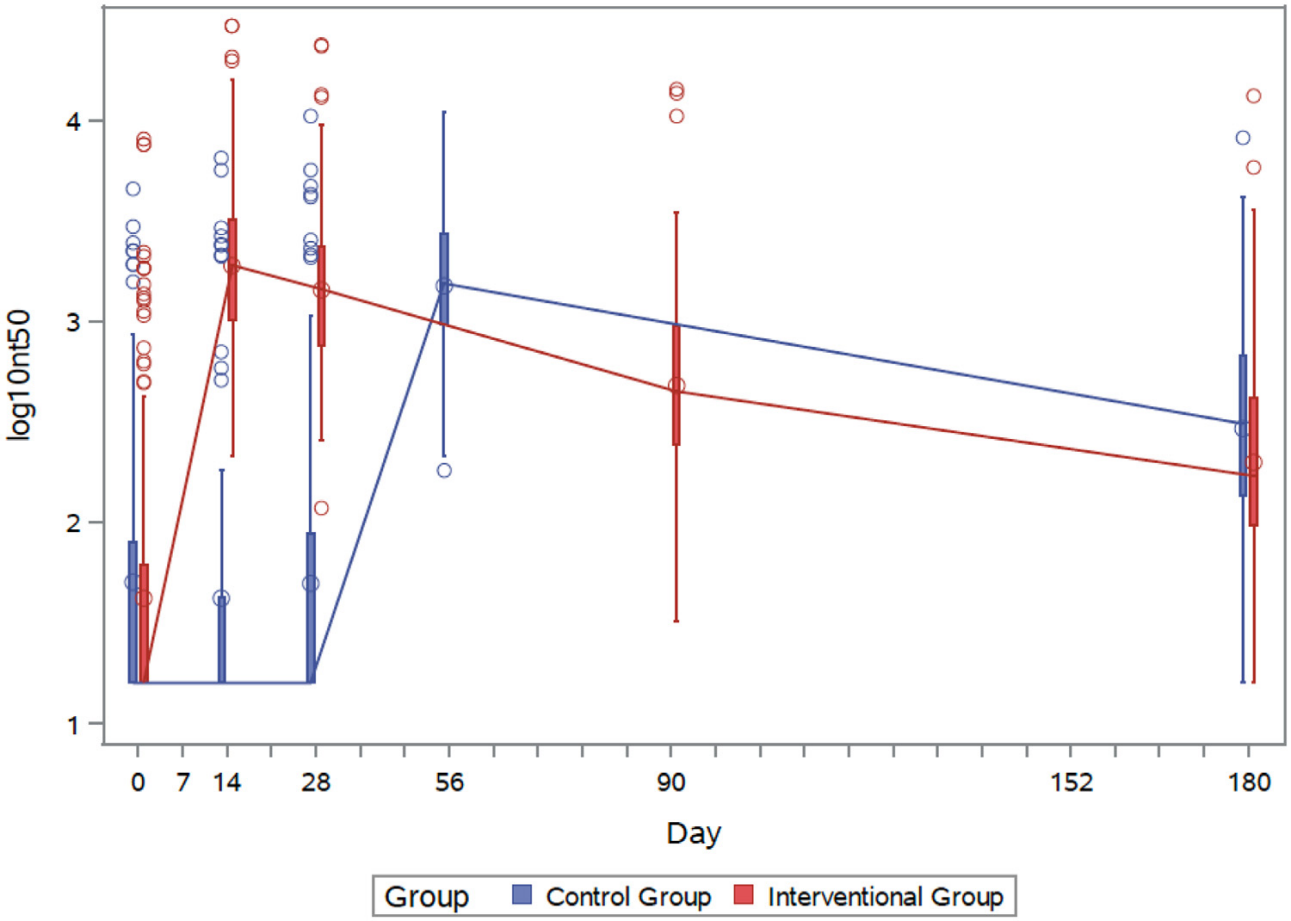
Neutralising antibodies titres (NT50) measured in both interventional (red) and control (blue) groups over time. Interventional group was immunised at day 0 and control group at day 28. Accordingly, day 180 corresponds to day 152 post vaccine in the control group.

Consistently with waning of antibody titres observed in the interventional group, a decay in neutralising antibodies was also evidenced. At day 14, GMT of neutralising antibodies was 1905·69 (95%CI 1625·65–2233·98) in the interventional group, which decreased to 1429·01 (95%CI 1220·37–1673·33) at day 28, to 480·68 (95%CI 398·27–580·13) at day 90 and to 198·72 (95%CI 161·54–244·47) at day 180. In the control group, neutralising antibody titres were similar to the interventional group both 28 days after second dose (1503·28 [95%CI 1210·71–1866·54]) and at day 180 (295·57 [95%CI 209·84–416·33]) (Figure 3; Appendix 1 p 7). RCDC for neutralising antibodies in both study groups are shown in Appendix 1 (p 8). All patients from both interventional and control group exhibited high neutralising activity (NT50 >1:100) against the reference variant G614 28 days post- BNT162b2 dose; a threshold that has been recently described^24^ as associated with vaccine efficacy. Yet decreased, a relevant proportion of individuals (76%) exhibited NT50 >1:100 at day 180 of study. (Appendix 1 p 9).

**Figure 4:**
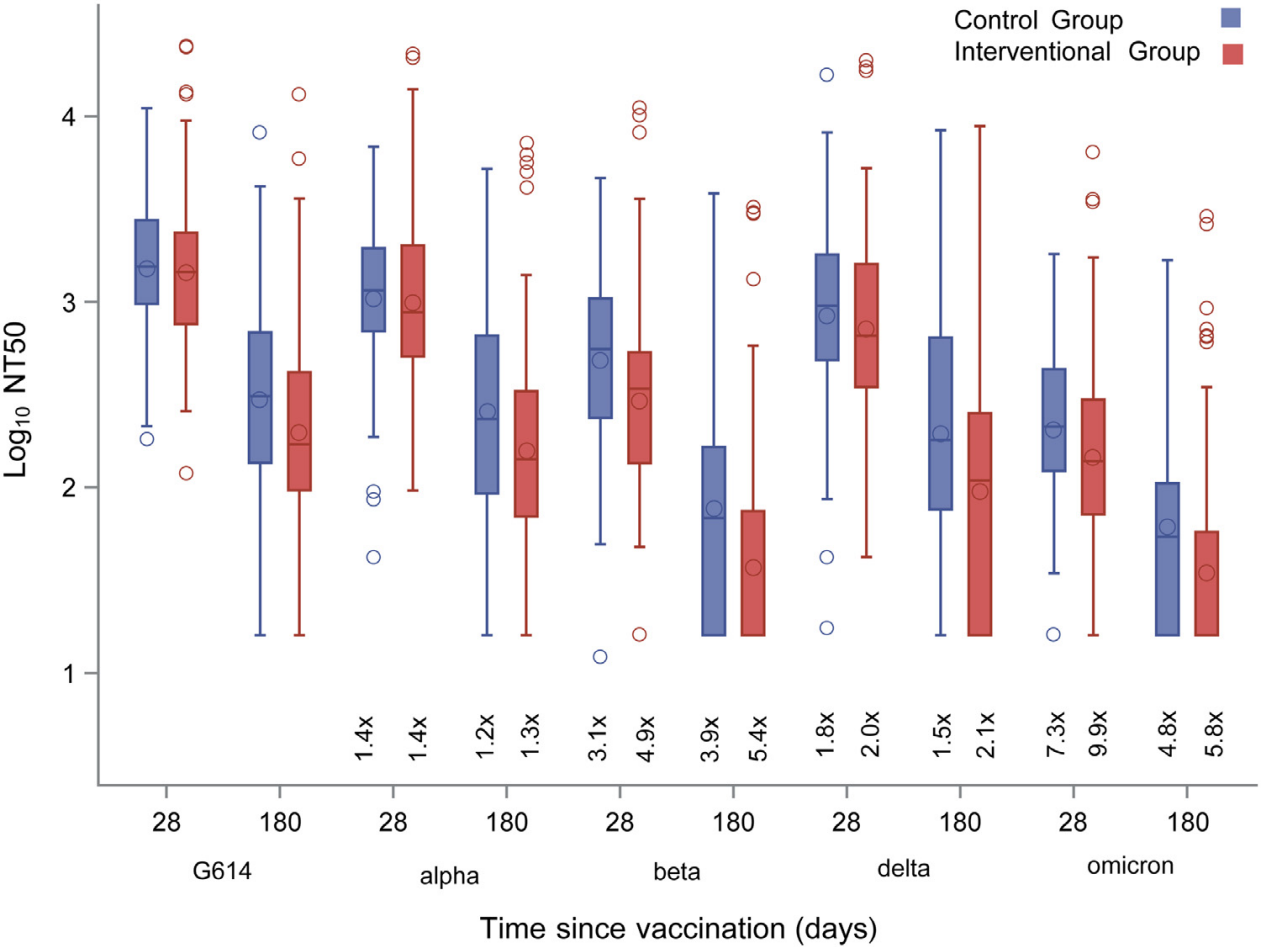
Neutralising antibodies titres (NT50) against SARS-CoV-2 variants measured in both interventional (red) and control (blue) groups at days 28 and 180 after BNT162b2 administration. Interventional group was immunised at day 0 and control group at day 28. Accordingly, day 180 corresponds to day 152 post vaccine in the control group. Dashes and circles inside boxes indicated the median and arithmetic mean, respectively. Box limits indicate the interquartile range (IQR). Whiskers are adjusted to maximal and minimal values if lower than 1.5 times the IQR. Further outliers are indicated as circles.

Differences between variants and original G614 strain at different time points were analysed (Appendix 1, p 10). By SARS-CoV-2 variants, the poorest neutralisation capability at day 28 post-vaccination was found with Omicron variant in both interventional and control groups (GMT 144·84 [95%CI 116·65–179·85] and 204·84 [95%CI 151·99–276·06]). A decrease in NT50 was also observed for Beta variant in both interventional and control groups (GMT 293·21 [95%CI 234·8–366·15] and 483·89 [95%CI 352·53–664·2]). Of note, titre of Omicron and Beta-neutralising antibodies at day 28 in the control group was higher than in the interventional group (*p* = 0·0641 and *p* = 0·0102, respectively), whilst no differences between groups were evidenced for Alpha- and Delta-neutralising antibody titres (Figure 4; Appendix 1 p 11). NT50 against Delta variant on day 28 after second dose was 717·13 (95%CI 587·27–876·13) in the interventional group and 837·14 (95%CI 609·7–1149·41) in the control group. Overall, day-28 NT50 was above 1:100 in 94% to 100% patients against all variants excepting Beta (88% patients) and Omicron (69%) (Appendix 1 p 12).

At day 180 Omicron-neutralising antibody titres decayed to 34·46 (95%CI 27·72–42·85) and 61·52 (95%CI 43·66–86·71) in the interventional and control groups, respectively (*p* = 0·0038). Beta-neutralising antibody titres decayed to 37·08 (95%CI 29·76–46·2) and 76·24 (95%CI 53·99–107·67) in the interventional and control groups, respectively (*p* = 0·0004). Delta NT50 decayed to 94·57 (95%CI 71·83–124·52) and 192·89 (95%CI 126·93–293·12) at day 180 in the interventional and control groups, respectively (*p* = 0·0043) (Figure 4; Appendix 1 p 11).

**Figure 5:**
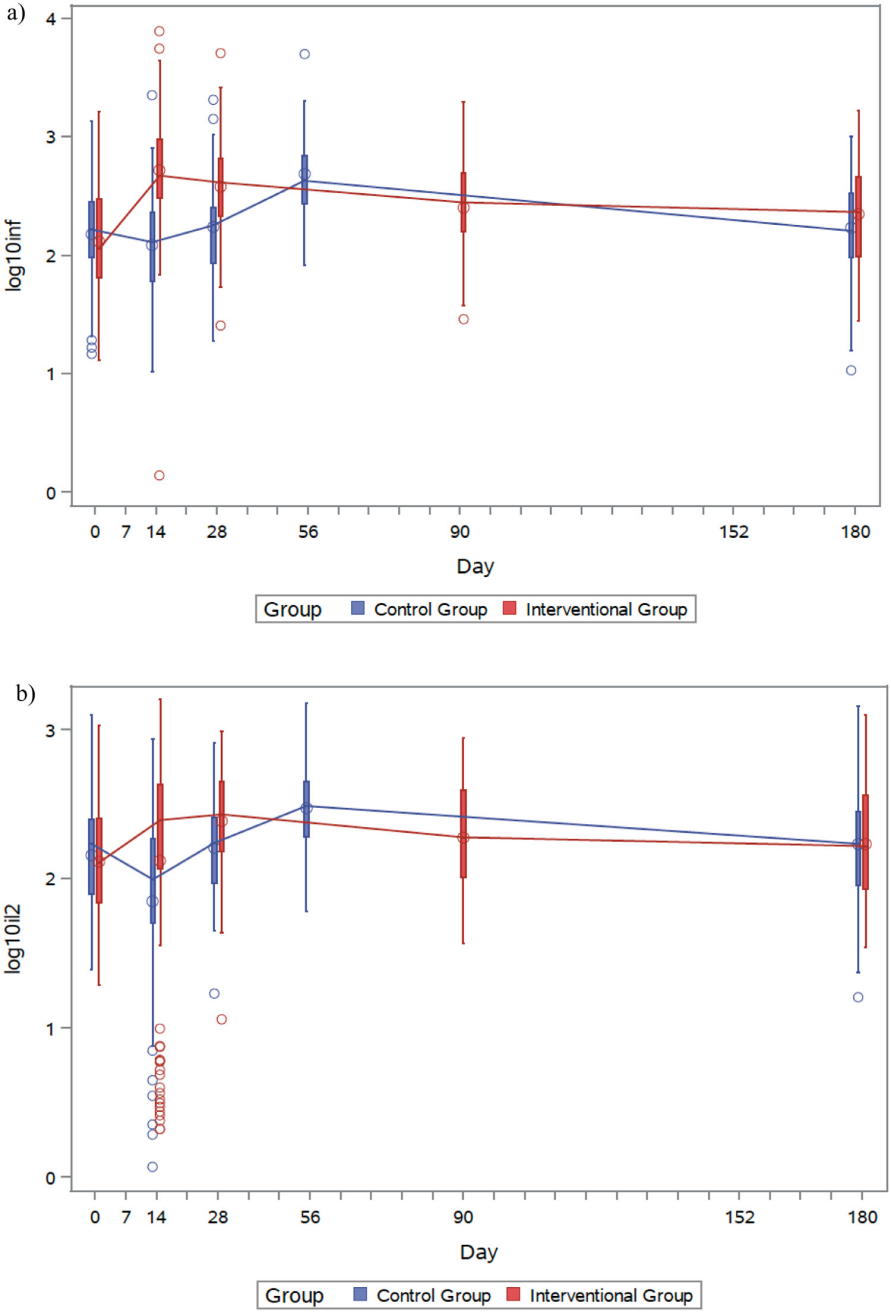
IFN-γ (a) and IL-2 (b) measured in both interventional (red) and control (blue) groups over time. Interventional group was immunised at day 0 and control group at day 28. Accordingly, day 180 corresponds to day 152 post vaccine in the control group.

Regarding dynamics of functional spike-specific T-cell response, an increase in levels of both IFN-γ and IL-2 after vaccination is followed by a progressive waning over time. In the interventional group, maximum IFN-γ production was observed at day 14 post-dose. Levels decreased to 380·93 pg/mL (95% CI 309·07–469·5) at day 28 and 223·8 pg/mL (166·25–301·28) at day 180. In the control group, IFN-γ levels 28 days after BNT162b2 dose were 485·32 pg/mL (343·51–685·68), and decreased to 171·23 pg/mL (120·15–244·02) at day 180. Similarly, IL-2 concentrations were maximum at day 28 post-vaccination in both the interventional and control groups (244·07 pg/mL [95% CI 204·89–290·74] and 299·2 [217·81–411·01], respectively) and progressively decayed until day 180 (171·54 [133·08–221·12] and 170·25 [122·24–237·11], respectively). Of note, day-180 levels of both IFN-γ and IL-2 were higher than those present at baseline (Figure 5; Appendix 1 p 13).

## Discussion

Our results provide evidence that humoral immune response of patients vaccinated with the heterologous ChAdOx1-S/BNT162b2 regime decays over time after peaking at day 14 post- BNT162b2 dose. A decline ranging 25%-27% in total RBD and neutralising antibody levels was observed after 28 days, which increased up to 70%-75% on day 90 and 86%-90% on day 180. This waning of immunogenicity was expected consistently with previous reports from COVID-19 mRNA vaccines^5^^,^^25^^,^^26^ that reported antibody half-life of 28-33 days.^26^ A similar decline of about 80% in antibody levels was found with homologous BNT162b2 regime at 90 days after the second dose^5^ as well as with homologous mRNA-1273 regime, albeit the latter to a lesser extent (decline around 60%).^25^ With homologous vaccination with ChAdOx1, antibodies are induced at lower levels than with homologous RNA regimens or heterologous vaccination of ChAdOx1 with BNT162b2^27^ or mRNA-1273,^28^ although it has been reported that antibodies decay with a slower kinetics.^29^ Furthermore, these results are consistent with known kinetics of humoral immune response against acute viral infections, in which extrafollicular short-lived plasmablasts contribute to early antibody production – IgM, IgG, IgA –, while a secondary increasing contribution of germinal centre-derived plasma cells – with longer lifespan and larger secretory capacity – leads to secretion of class-switched antibodies, mainly IgG. Considering that half-life of IgM is substantially shorter than IgG, a decay in antibody titres is common once the extrafollicular response is resolved. However they will rapidly rise if memory B cells are re-exposed to viral antigens in the future.^30^ In this regard, we observed a slowing-down in SARS-CoV-2 antibody decay from month 3 to month 6, consistent with previous reports.^26^

BNT162b2 administration to the control group 28 days later than the interventional one did not result in worse or weaker antibody responses 28 days after immunization. Actually, S-RBD antibodies and all variant-specific neutralising titres were higher – S-RBD and Beta-specific titres significantly higher– in the control group four weeks after vaccination suggesting a benefit of second dose delay. However, lack of antibody determination 14 days after immunization – when top levels of antibodies are reached – does not allow to perform a parallel kinetics of S-RBD and neutralizing antibodies between CG and IG to fully demonstrate that delayed administration of BNT162b2 results in better antibody responses.

Importantly, our results suggest that high levels of protection against Delta-variant persisted in both IG and CG at day 28 after the second dose of the heterologous ChAdOx1-S/BNT162b2 scheme.

Regarding differences found at day 180 between control and intervention group it must be noted that this measurement carried over the 28-day delay in BNT162b2 administration to the control group (measured at day 152 – instead of 180 – after dose). A second explanation for these differences could be related with vaccination delay itself, supporting an apparent benefit for longer intervals between doses as found previously.^14^^,^^31^ Indeed, results from regression models pointed in this direction. Such delay could favour the maturation process of memory B cells from germinal centres (GC), over which B cells accumulate somatic mutations in their variable region leading to selection of those with higher affinity for a given viral antigen.^30^ A recent study has demonstrated that antigen-driven activation of memory B cells persisted and matured up to 6 months after SARS-CoV-2 infection.^32^ The observation that at day 28 neutralisation activity in the control group against Beta was higher – and trended to higher against Omicron – while no differences were observed between groups for G614 reference strain, Alpha and Delta variants suggests that the 4-week delay in vaccination window for the control group in our study might have contributed to a better affinity maturation against ‘difficult’ variants such as Beta and Omicron. Regarding cellular responses, similar decay of IFN-γ and IL-2 were found in both groups at different time points which is consistent with the generation of memory T lymphocytes in which maturation process and selection of Tc receptor affinity is not dependent on somatic mutation.

Notwithstanding this, the sharp decay in RBD antibodies and neutralisation titres observed at day 180 as compared to day 14 and 28 support the use of a third immunization to reach higher protection levels, particularly considering the high infectivity potential of the Omicron variant.^33^ Actually, despite the persistence of immune memory, antibody decay increases the risk of SARS-CoV-2 infection and a third dose becomes necessary to achieve protection against asymptomatic and symptomatic infections, particularly in aged groups above 60 and patients with risk factors for developing severe COVID-19 as immune suppression.^19^^,^^20^ It has been described that boosting with a third dose of mRNA vaccines generate potent neutralization of Omicron, shortening the difference in neutralization titres with other variants.^34^ Thus, increased protection of a booster dose would be related not only to higher levels of neutralizing antibodies but to antibody maturation leading to the generation of antibodies with increased affinity to their targets. These qualitative changes are particularly important against escape variants as Omicron and represent an added value for a third dose. Unfortunately, very recently it has been described a waning effectiveness of a third dose of BNT162b2 against hospital admission after 3 months due to the Omicron variant.^35^

As previously described,^1^ the main limitation of the CombiVacS study is the absence of a control group completing the homologous ChAdOx1-S scheme to compare with the heterologous ChAdOx1-S/BNT162b2 regimen. This arm would had been very useful to compare antibody waning and neutralization activity in individuals vaccinated with homologous or heterologous vaccine regimes. Besides, the abovementioned 4-week delay between both study groups in BNT162b2 administration led to capture 5-month, rather than 6-month, post dose data in the control group. Although this limitation may have influenced some differences observed at day 180, overall results are consistent between groups. As mentioned, lack of antibody determination at day 14 in the CG limits the interpretation of the results. Also, we have found a low proportion of high-responder outliers, in particular before immunization with the second vaccine dose. We cannot rule out asymptomatic SARS-CoV-2 infection between both vaccine doses leading to a “booster-like” response after first immunization.

In conclusion, follow-up of individuals included in the CombiVacS trial that were immunised with heterologous ChAdOx1-S/BNT162b2 confirm waning of humoral and cellular responses over time, nevertheless a relevant proportion of individuals exhibited neutralising activity > 1:100 six months after full vaccination excepting against Beta and Omicron variants. These results support the use of a third dose six months after regular vaccination to enhance immune response, particularly against new VoCs, as Omicron. Further studies addressing immunogenicity using different heterologous vaccination schemes are warranted.

## Contributors

Trial conceptualisation was done by C.B.-I., J.A., M.P.-O., A.M.B., A.J.C., J.F., J.R.A. and M.C. A.J.C., J.F., and A.Ag. developed the study methods. J.A., M.P.-O., A.M.B., J.F., L.C., M.J.B., J.G.-P., M.C., A.P., M.G.-P., E.A.-A., M.T., A.As., N.I.-A., E.M., C.P.-I., J.O., M.C.-O., M.B., P.C., L.H.-G., I.F., H.E.D.T. and J.R.A. were study investigators. M.T.G.M., D.L., J.G.-P. and A.G.C. ensured data verification. J.G.-P., D.L., J.A., M.P.-O., M.T.G.M., J.O., A.M.B. and A.J.C. were responsible for the present secondary statistical analysis. C.B.-I., J.A., M.P.-O., J.G.-P., M.G.-P., A.M.B., L.C., M.C., M.J.B., A.P., J.O., J.F., and J.R.A. supervised the study. C.B.-I. was responsible for funding acquisition. J.A., M.P.O., J.O., J.G.-P. and A.M.B. wrote the original draft of this Article. All authors reviewed and edited the manuscript, and approved the manuscript for submission. All authors reviewed and approved the original draft. All authors had full access to the full data in the study and accept responsibility to submit for publication.

## Data sharing statement

Individual participant data will be made available when the trial is complete, on request to the corresponding authors. After approval of a proposal, data will be shared through a secure online platform.

## Declaration of interests

JA has received fees for educational programs from Gilead, MSD, GSK and Janssen outside of the submitted work. MC has participated in advisory boards and has received research funding from GSK, Sanofy Pasteur, Pfizer, Novavax and Janssen.CB-I is the deputy general manager of the Instituto de Salud Carlos III. JRA has received fees from Janssen, outside of the submitted work. AMB is principal investigator of clinical trials sponsored by GlaxoSmithKline, Daiichi-Sankyo, Janssen, and Farmalider, outside of the submitted work. All other authors declare no competing interests.

## Figures and Tables

**Table:** 

FIST NAME	SURNAME
Isabel Concepción Giovanni Javier María Jesús Francisco Esther Almudena Paloma Jana Isabel Marcos Pilar	Jado Perea Fedele Hernández Llamas Díez-Fuertes Calonge Cascajero Jiménez-Santana Baranda Cervera Berges-Buxeda Portolés
Lucía Amelia Lucía Elena Enrique Stefan Mark Alicia Irene Mikel Jaime Paula Marta Cristina Vega Raquel Laura Julio Antonio Eduardo Carmen Esther Pilar María Victoria Paloma Amparo Rocío Silvia Cristina Marina Fernando Marta Blanca Susana Aitor Ana Mikel Dolores Ana Belén Gustavo Olaia Josu Maria Angeles Alejandro Inés Rosa Begoña Laura Sara Marta Lourdes E Mª Ángeles	Martínez de Soto Rodríguez Mariblanca Díaz García Ramírez García Seco Meseguer Stewart Balbás Marín Candón García García Urroz Elizalde Monserrat Villatoro de la Rosa Sanz García López Crespo Mauleón Martínez de Madariaga Castell Vitón Vara García Rodríguez Buño López Granados Cámara Rey Cuevas Ayllon García Jiménez González Hernández Rubio Moraga Alapont Sánchez Prieto Llorente Gómez Miragall Roig Aparicio Marlasca de la Calle Arsuaga Duque Meijide García de Vicuña San Expósito Gallego García-Vázquez de la Hoz Pérez-Nanclares Velasco Aurrekoetxea Lázaro García Castaño Urrutia Martínez Calvo Saso Gómez Aldea Barón-Mira Marcos
Laura Sulayman Sara Montserrat Sebastiana Anna Anna Victoria Antoni Begoña Elisenda Sheila Francisco Javier Cristina Laura Juan José Patricia Josep Lluís Jordi Xavier Susana Blanca Oleguer Cesar José Angel Lluis Sonia Judit José Carla Lina Aitana Carla Laia Gisela Carmen Gloria Esther Margarita Ana Julia Elena Ballarin Eulàlia Lourdes Leonor Manuel Jose Antonio Esperanza Natalia Ana Belén Teresa Ouhao	Granés Lazaar Herranz Malet Quesada Vilella Llupià Olivé Trilla Gómez González Romero Gámez Casals Burunat Castelló Fernández Bedini Vila Martínez-Gómez Otero-Romero Borras-Bermejo Parés-Badell Llorente Rodrigo-Pendás Armadans Uriona Riera-Arnau Santos Sans-Pola Camacho-Arteaga Plaza Aguilar Pinos Gili Altadill Torres Palacio Torrens Feliu Calonge Ballarín Alins Pérez-Esquirol Vendrell Bosch Laredo Sanchez-Craviotto Gil-Marin Gonzalez-Rojano Rodriguez-Galán Rivas-Paterna Iglesias Zhu-Huang
Angel Daniel Verónica Oliver Natalia María Aránzazu Carmen Agustín	Hernández-Bartolomé Lozano-Martín Alvarez-Morales Astasio Pérez-Macías Urrutia-de-la-Plaza Sanz Molina

## Appendix B Supplementary materials

Image, application 1

Image, application 2
