# Supplementary material for: Immunogenic dynamics and SARS-CoV-2 variant neutralisation of the heterologous ChAdOx1-S/BNT162b2 vaccination: Secondary analysis of the randomised CombiVacS study
Source: eClinicalMedicine. 2022 Jul 1;50:101529. doi: 10.1016/j.eclinm.2022.101529 (PMC9249304; doi:10.1016/j.eclinm.2022.101529)
Supplement: Supplementary file 2 [file mmc2.docx]

**SUPPLEMENTARY APPENDIX 1**

Supplementary tables and figures 2

Figure S1. Graphic and statistics of day-28 post-BNT162b2 blood sample collection 2

Table S1. Summary statistics for SARS-CoV-2 anti-spike antibody response related to BNT162b2 administration time (day 0) 3

Figure S2. Reverse cumulative distribution curves for RBD anti-spike antibodies from day 0 to day 180 in the interventional group (a) and the control group (b) 4

Table S2. Summary statistics for SARS-CoV-2 anti-spike antibody response related to BNT162b2 administration time (day 0) stratified by sex 5

Table S3. Summary statistics for SARS-CoV-2 anti-spike antibody response related to BNT162b2 administration time (day 0) stratified by age 6

Table S4. Summary statistics for neutralising antibody response related to BNT162b2 administration time (day 0) 7

Figure S3. Reverse cumulative distribution curves for neutralising antibodies over time in the interventional (a) and control (b) groups 8

Table S5. Neutralising activity (NT50 G614) by categories 9

Table S6. Comparison of neutralizing activity between variants and the original G614 at different time points……………………………………………………………………………………………………………………………………………….. 10

Table S7. Statistics for neutralising antibody response by SARS-CoV-2 variants 11

Table S8. Neutralising activity (NT50) by categories and SARS-CoV-2 variants 12

Table S9. Summary statistics for cellular immune response related to BNT162b2 administration time (day 0) 13

Figure S4. Plot of Total Sample Size versus Power for Two-Sample t-test for Mean Ratio 14

Table S10. Effect coefficient of interventional group versus control group adjusted by sex, age, and time between vaccine doses (model 1) and adjusted by sex, age, time between vaccine doses, comorbidities and BMI (model 2). 15

Table S11. Baseline characteristics of the groups under comparisons at day 180 16

Supplementary Methods 17

Table S12. Number of subjects with full data for each variable analysed 17

Pseudo-virus neutralisation assays 18

Cellular immunity quantification 19

Supplementary Author List 20

CombiVacS Vaccine Trial Group 20

Data and Safety Monitoring Board (DSMB) 23

Steering Committee (SC) 24

**Study Protocol…………………………………………….…………………………………………..……………………………….26**

# Supplementary tables and figures

## Figure S1. Graphic and statistics of day-28 post-BNT162b2 blood sample collection


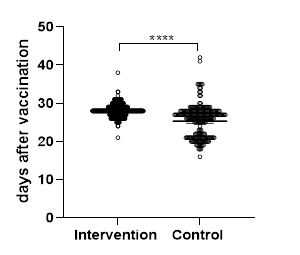


**** p<0·0001

|  | **Interventional group** | **Control group** |
| --- | --- | --- |
| Number of values | 441 | 210 |
| Minimum, days | 21 | 16 |
| 25% Percentile, days | 27 | 21·75 |
| Median, days | 28 | 27 |
| 75% Percentile, days | 29 | 28 |
| Maximum, days | 38 | 42 |
| Range, days | 17 | 26 |
| 95% CI of median | | |
| Actual confidence level | 95·46% | 95·49% |
| Lower confidence limit | 28 | 26 |
| Upper confidence limit | 28 | 27 |
| Mean | 28·1 | 25·67 |
| Standard Deviation | 1·558 | 4·125 |
| Standard Error of Mean | 0·07419 | 0·2847 |
| Lower 95% CI of mean | 27·95 | 25·11 |
| Upper 95% CI of mean | 28·24 | 26·23 |

## Table S1. Summary statistics for SARS-CoV-2 anti-spike antibody response related to BNT162b2 administration time (day 0)

|  | **N** | **Interventional group,**  **geometric mean [95% CI]** | **N** | **Control group,**  **geometric mean [95% CI]** | **Ratio [95% CI]** | **p-value** |
| --- | --- | --- | --- | --- | --- | --- |
| RBD day -28 |  | - | 223 | 91·33[69·42;120·17] | - | - |
| RBD day -21 |  | - | 222 | 91·68[70·32;119·52] | - | - |
| RBD day -14 |  | - | 223 | 101·56[78·17;131·94] | - | - |
| RBD day 0 | 444 | 71·07[59·58;84·78] | 219 | 103·08[79·01;134·47] | 0·69[0·5;0·94] | 0·0197 |
| RBD day 7 | 443 | 4327·63[3829·07;4891·11] |  | NA | - | - |
| RBD day 14 | 444 | 7739·21[7356·77;8141·53] |  | NA | - | - |
| RBD day 28 | 439 | 5616·91[5296·49;5956·71] | 212 | 7298·22[6739·41;7903·37] | 0·77[0·7;0·85] | <0·0001 |
| RBD day 90 | 425 | 2303·28[2141·66;2477·1] |  | NA | - | - |
| RBD day 180* | 416 | 1142[1048·69;1243·62] | 198 | 1836·4[1621·62;2079·62] | 0·62[0·54;0·72] | <0·0001 |

Units: BAU/mL

* Corresponded to day 152 post-dose in the control group. NA: not available (titres at days 7 and 14 post- BNT162b2 not planned in the control group; titres at day 90 post-dose not planned in the control group)

## Figure S2. Reverse cumulative distribution curves for RBD anti-spike antibodies from day 0 to day 180 in the interventional group (a) and the control group (b)

2a)


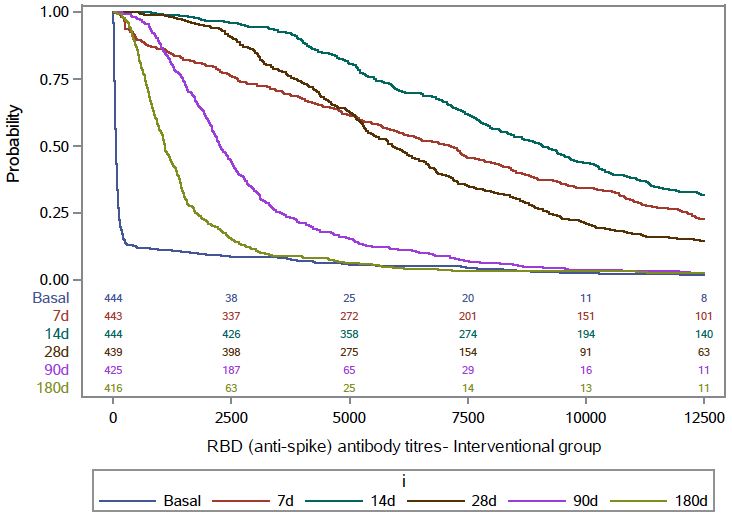


2b)


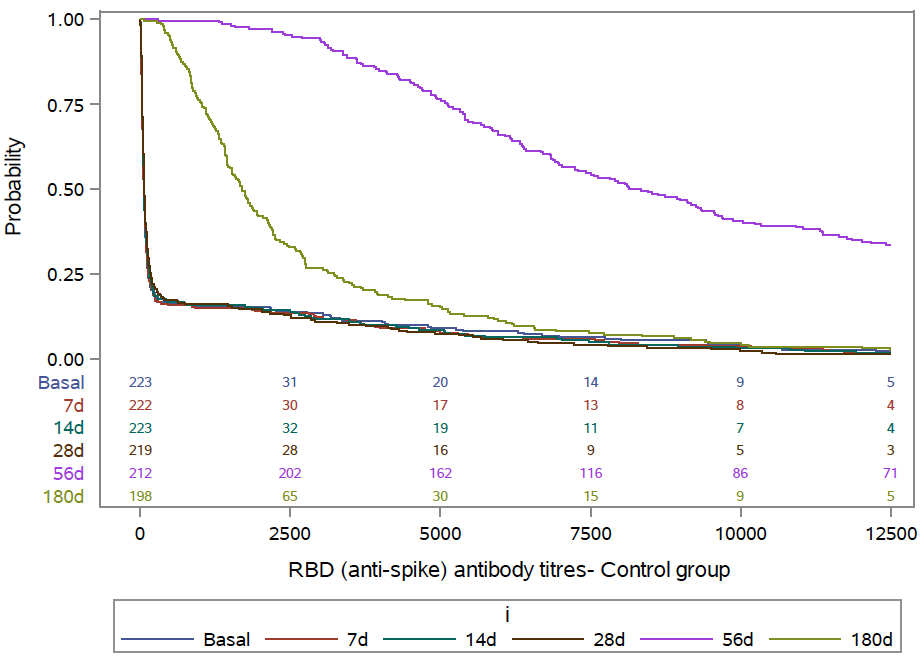


## Table S2. Summary statistics for SARS-CoV-2 anti-spike antibody response related to BNT162b2 administration time (day 0) stratified by sex

|  | **N** | **Interventional group,**  **geometric mean [95% CI]** | **N** | **Control group,**  **geometric mean [95% CI]** | **Ratio [95% CI]** | **p-value** |
| --- | --- | --- | --- | --- | --- | --- |
| **MALE** | | | | | | |
| RBD day -28 |  | - | 99 | 100·56[64·31;157·24] | - | - |
| RBD day -21 |  | - | 99 | 97·75[63·26;151·03] | - | - |
| RBD day -14 |  | - | 99 | 111·09[73·2;168·61] | - | - |
| RBD day 0 | 190 | 61·64[46·96;80·9] | 97 | 109·16[72·01;165·47] | 0·56 [0·35;0·91] | 0·0200 |
| RBD day 7 | 190 | 3382·1[2736·33;4180·27] |  | NA | - | - |
| RBD day 14 | 190 | 7197·64[6665·03;7772·82] |  | NA | - | - |
| RBD day 28 | 186 | 5170·36[4741·42;5638·12] | 91 | 6939·27[6033·28;7981·31 | 0·75 [0·64;0·87] | 0·0003 |
| RBD day 90 | 177 | 2220·41[1990·58;2476·77] |  | NA | - | - |
| RBD day 180* | 175 | 1180·59[1029·27;1354·16] | 84 | 1976·92[1605·94;2433·6] | 0·6[0·47;0·76] | <0·0001 |
| **FEMALE** | | | | | | |
| RBD day -28 |  | - | 124 | 84·58[59·85;119·53] | - | - |
| RBD day -21 |  | - | 123 | 87·07[62·49;121·31] | - | - |
| RBD day -14 |  | - | 124 | 94·54[67·47;132·47] | - | - |
| RBD day 0 | 254 | 79·06[62·68;99·73] | 122 | 98·48[69·45;139·64] | 0·8 [0·53;1·21] | 0·2952 |
| RBD day 7 | 253 | 5207·79[4524·74;5993·96] |  |  |  |  |
| RBD day 14 | 254 | 8170·79[7640·89;8737·44] |  |  |  |  |
| RBD day 28 | 253 | 5969·61[5514·83;6461·9] | 121 | 7580·36[6907·31;8319] | 0·79 [0·7;0·89] | 0·0001 |
| RBD day 90 | 248 | 2364·32[2144·07;2607·18] |  |  |  |  |
| RBD day 180* | 241 | 1114·77[999·6;1243·21] | 114 | 1739·28[1490·44;2029·68] | 0·64 [0·53;0·78] | <0·0001 |

Units: BAU/mL

* Corresponded to day 152 post-dose in the control group. NA: not available (titres at days 7 and 14 post- BNT162b2 not planned in the control group; titres at day 90 post-dose not planned in the control group).

## Table S3. Summary statistics for SARS-CoV-2 anti-spike antibody response related to BNT162b2 administration time (day 0) stratified by age

|  | **N** | **Interventional group,**  **geometric mean [95% CI]** | **N** | **Control group,**  **geometric mean [95% CI]** | **Ratio [95% CI]** | **p-value** |
| --- | --- | --- | --- | --- | --- | --- |
| **18-49 YEARS** | | | | | | |
| RBD day -28 |  | - | 141 | 92·51[65·69;130·28] | - | - |
| RBD day -21 |  | - | 141 | 92·19[66·65;127·53] | - | - |
| RBD day -14 |  | - | 141 | 99·42[71·74;137·79] | - | - |
| RBD day 0 | 290 | 68·19[55·24;84·18] | 138 | 103·02[74·17;143·09] | 0·66 [0·45;0·97] | 0·0330 |
| RBD day 7 | 290 | 4499·01[3906·02;5182·03] |  | NA | - | - |
| RBD day 14 | 290 | 7616·85[7136·28;8129·78] |  | NA | - | - |
| RBD day 28 | 286 | 5438·31[5047·1;5859·84] | 133 | 7408·33[6782·33;8092·11] | 0·73 [0·65;0·82] | <0·0001 |
| RBD day 90 | 274 | 2185·04[1991·52;2397·37] |  | NA | - | - |
| RBD day 180* | 270 | 1084·51[972·16;1209·83] | 121 | 1768·42[1521·06;2056] | 0·61[0·51;0·74] | <0·0001 |
| **50-59 YEARS** | | | | | | |
| RBD day -28 |  | - | 82 | 89·34[55·94;142·68] | - | - |
| RBD day -21 |  | - | 81 | 90·79[56·92;144·81] | - | - |
| RBD day -14 |  | - | 82 | 105·34[67·38;164·71] | - | - |
| RBD day 0 | 154 | 76·83[55·73;105·91] | 81 | 103·17[65·1;163·51] | 0·74 [0·43;1·29] | 0·2933 |
| RBD day 7 | 153 | 4020·5[3181·95;5080·03] |  | NA | - | - |
| RBD day 14 | 154 | 7974·98[7361·48;8639·61] |  | NA | - | - |
| RBD day 28 | 153 | 5966·64[5426·57;6560·46] | 79 | 7116·54[6086·62;8320·73] | 0·84 [0·71;1] | 0·0450 |
| RBD day 90 | 151 | 2534·42[2255·71;2847·57] |  | NA | - | - |
| RBD day 180* | 146 | 1256·48[1098;1437·84] | 77 | 1948·53[1565·62;2425·11] | 0·64[0·51;0·82] | 0·0005 |

Units: BAU/mL

* Corresponded to day 152 post-dose in the control group. NA: not available (titres at days 7 and 14 post- BNT162b2 not planned in the control group; titres at day 90 post-dose not planned in the control group)

## Table S4. Summary statistics for neutralising antibody response related to BNT162b2 administration time (day 0)

|  | **N** | **Interventional group,**  **geometric mean [95% CI]** | **N** | **Control group,**  **geometric mean [95% CI]** | **Ratio [95% CI]** | **p-value** |
| --- | --- | --- | --- | --- | --- | --- |
| NT50 day -28 |  | - | 69 | 50·84 [33·56;76·99] | - | - |
| NT50 day -14 |  | - | 69 | 41·81 [27·18;64·32] | - | - |
| NT50 day 0 | 129 | 41·84 [31·28;55·96] | 67 | 49·93 [30·76;81·06] | 0·84 [0·49;1·42] | 0·5113 |
| NT50 day 14 | 129 | 1905·69 [1625·65;2233·98] | - | NA | - | - |
| NT50 day 28 | 127 | 1429·01 [1220·37;1673·33] | 66 | 1503·28 [1210·71;1866·54] | 0·95[0·73;1·24] | 0·7088 |
| NT50 day 90 | 121 | 480·68 [398·27;580·13] | - | NA | - | - |
| NT50 day 180* | 119 | 198·72 [161·54;244·47] | 59 | 295·57 [209·84;416·33] | 0·67 [0·46;0·98] | 0·0394 |

* Corresponded to day 152 post-dose in the control group. NA: not available (titres at days 7 and 14 post- BNT162b2 not planned in the control group; titres at day 90 post-dose not planned in the control group)

## Figure S3. Reverse cumulative distribution curves for neutralising antibodies over time in the interventional (a) and control (b) groups

**a)**

**
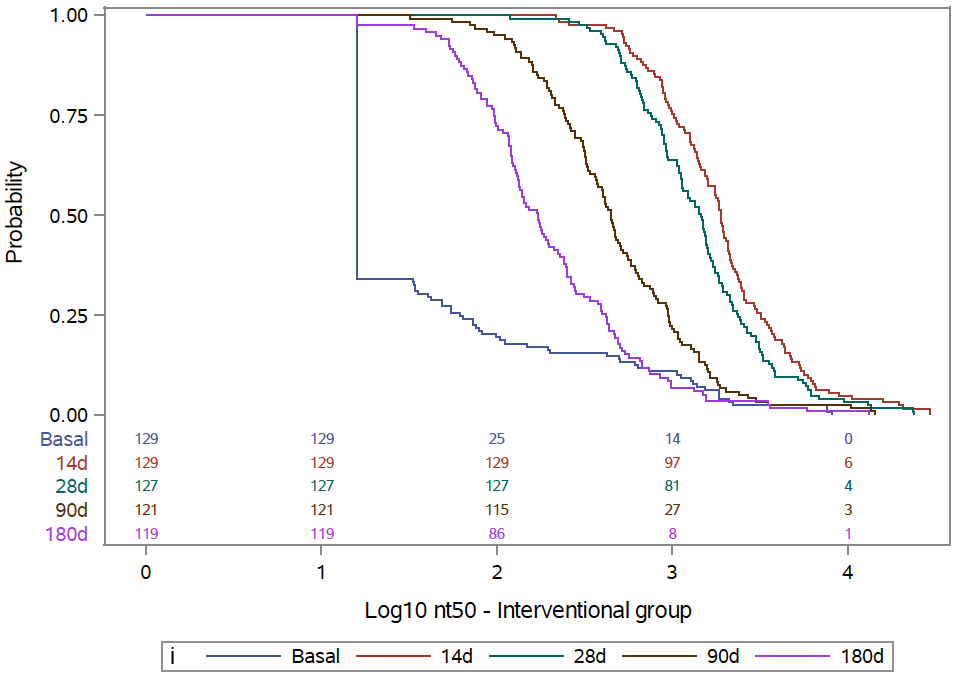
**

**b)**

**
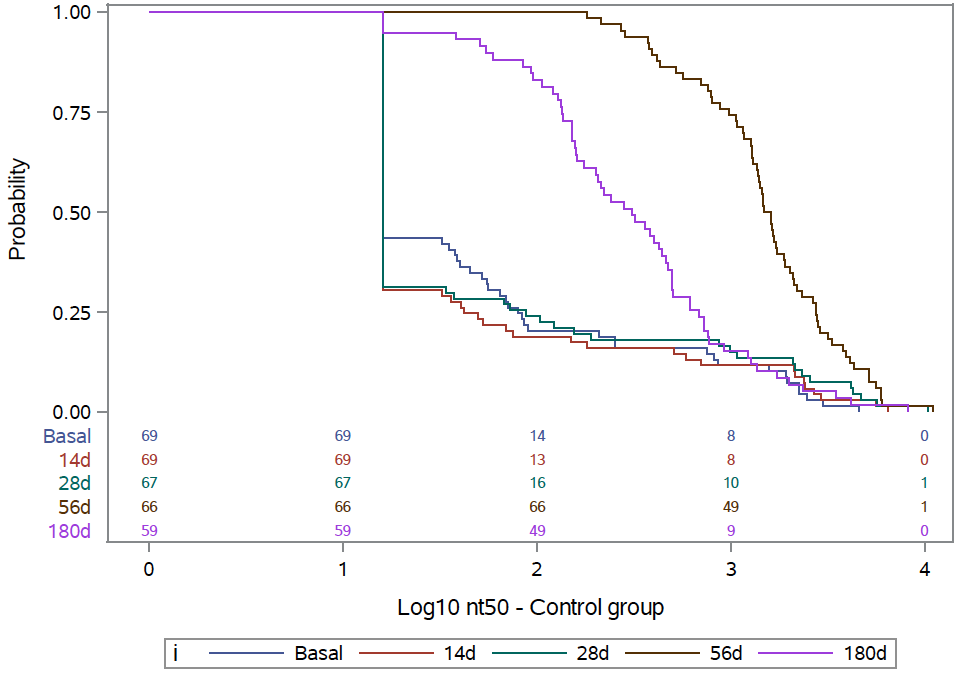
**

## Table S5. Neutralising activity (NT50 G614) by categories

|  | **Interventional group (n=129)** | | | | **Control group (n=69)** | | |
| --- | --- | --- | --- | --- | --- | --- | --- |
|  | **Day 0**  **(n= 129)** | **Day 28 post-** **BNT162b2**  **(n= 127)** | **Day 90 post-** **BNT162b2**  **(n= 121)** | **Day 180 post-** **BNT162b2**  **(n= 119)** | **Day 0**  **(n= 67)** | **Day 28 post-** **BNT162b2**  **(n= 66)** | **Day 152 post-** **BNT162b2**  **(n= 59)** |
| **Negative (<32), n (%)** | 85 (65·9) | 0 (0) | 0 (0) | 3 (2·5) | 46 (66·7) | 0 (0) | 3 (5·1) |
| **>32 to <100, n (%)** | 11 (8·5) | 0 (0) | 6 (5·0) | 30 (25·2) | 5 (7·2) | 0 (0) | 7 (11·9) |
| **>100 to <300, n (%)** | 13 (10·1) | 3 (2·4) | 31 (25·6) | 50 (42·0) | 4 (5·8) | 4 (6·1) | 19 (32·2) |
| **>300 to <1000, n (%)** | 6 (4·7) | 43 (33·9) | 57 (47·1) | 28 (23·5) | 2 (2·9) | 13 (19·7) | 21 (35·6) |
| **≥1000, n (%)** | 14 (10·9) | 81 (63·8) | 27 (22·3) | 8 (6·7) | 10 (14·5) | 49 (74·2) | 9 (15·3) |

## Table S6. Comparison of neutralizing activity between variants and the original G614 at different time points

|  | | **Control group** | **Interventional group** |
| --- | --- | --- | --- |
| **Comparison between variants** | **Time** | **p-value** | **p-value** |
| G614 - Alpha | 28d | 0.1020 (0.0255) | 0.0132 (0.0033) |
|  | 180d | 0.9999 (0.5730) | 0.5548 (0.1387) |
| G614 – Beta | 28d | 0.0004 (0.0001) | 0.0004 (0.0001) |
|  | 180d | 0.0004 (0.0001) | 0.0004 (0.0001) |
| G614 – Delta | 28d | 0.0116 (0.0029) | 0.0004 (0.0001) |
|  | 180d | 0.4676 (0.1169) | 0.0004 (0.0001) |
| G614 – Omicron | 28d | 0.0004 (0.0001) | 0.0004 (0.0001) |
|  | 180d | 0.0004 (0.0001) | 0.0004 (0.0001) |

## Table S7. Statistics for neutralising antibody response by SARS-CoV-2 variants

|  | **N** | **Interventional group,**  **GMT [95% CI]** | **N** | **Control group,**  **GMT [95% CI]** | **Ratio [95% CI]** | **p-value** |
| --- | --- | --- | --- | --- | --- | --- |
| NT50 G614  day 28  post-BNT162b2 | 127 | 1429·01  [1220·37;1673·33] | 66 | 1503·28  [1210·71;1866·54] | 0·95  [0·73;1·24] | 0·7088 |
| NT50 Alpha  day 28 post-BNT162b2 | 127 | 987·41  [817·39;1192·8] | 66 | 1038·4  [812·68;1326·8] | 0·95  [0·69;1·3] | 0·7523 |
| NT50 Beta  day 28 post-BNT162b2 | 127 | 293·21  [234·8;366·15] | 66 | 483·89  [352·53;664·2] | 0·61  [0·41;0·89] | 0·0102 |
| NT50 Delta  day 28 post-BNT162b2 | 127 | 717·3  [587·27;876·13] | 66 | 837·14  [609·7;1149·41] | 0·86  [0·6;1·22] | 0·3942 |
| NT50 Omicron  day 28 post-BNT162b2 | 127 | 144·84  [116·65;179·85] | 66 | 204·84  [151·99;276·06] | 0·71  [0·49;1·02] | 0·0641 |
| NT50 G614  day 180 post- BNT162b2 | 119 | 198·72  [161·54;244·47] | 59 | 295·57  [209·84;416·33] | 0·67  [0·46;0·98] | 0·0394 |
| NT50 Alpha  day 180 post-BNT162b2 | 119 | 156·92  [123·8;198·9] | 59 | 256·35  [177·11;371·06] | 0·61  [0·4;0·93] | 0·0230 |
| NT50 Beta  day 180 post-BNT162b2 | 119 | 37·08  [29·76;46·2] | 59 | 76·24  [53·99;107·67] | 0·49  [0·33;0·72] | 0·0004 |
| NT50 Delta  day 180 post-BNT162b2 | 119 | 94·57  [71·83;124·52] | 59 | 192·89  [126·93;293·12] | 0·49  [0·3;0·8] | 0·0043 |
| NT50 Omicron  day 180 post-BNT162b2* | 119 | 34·46  [27·72;42·85] | 59 | 61·52  [43·66;86·71] | 0·56  [0·38;0·83] | 0·0038 |

* Day 152 post-BNT162b2 in the control group. GMT: geometric mean titre

## Table S8. Neutralising activity (NT50) by categories and SARS-CoV-2 variants

|  | **Day 28 post-BNT162b2** | | | | | | | | | | **Day 180 post-BNT162b2*** | | | | | | | | | |
| --- | --- | --- | --- | --- | --- | --- | --- | --- | --- | --- | --- | --- | --- | --- | --- | --- | --- | --- | --- | --- |
|  | **Interventional group (n= 127)** | | | | | **Control group (n= 66)** | | | | | **Interventional group (n= 119)** | | | | | **Control group (n= 59)** | | | | |
|  | **G614** | **Alpha** | **Beta** | **Delta** | **Omicron** | **G614** | **Alpha** | **Beta** | **Delta** | **Omicron** | **G614** | **Alpha** | **Beta** | **Delta** | **Omicron** | **G614** | **Alpha** | **Beta** | **Delta** | **Omicron** |
| **Negative (<32), n (%)** | 0 (0) | 0 (0) | 7 (5·5) | 0 (0) | 13 (10·2) | 0 (0) | 0 (0) | 2 (3) | 2 (3) | 6 (9·1) | 3 (2·5) | 11 (9·2) | 67 (56·3) | 34 (28·6) | 72 (60·5) | 3 (5·1) | 4 (6·8) | 16 (27·1) | 10 (16·9) | 21 (35·6) |
| **>32 to <100, n (%)** | 0 (0) | 1 (0·8) | 13 (10·2) | 5 (3·9) | 32 (25·2) | 0 (0) | 3 (4·5) | 6 (9·1) | 2 (3) | 9 (13·6) | 30 (25·2) | 31 (26·1) | 33 (27·7) | 24 (20·2) | 30 (25·2) | 7 (11·9) | 12 (20·3) | 22 (37·3) | 7 (11·9) | 21 (35·6) |
| **>100 to <300, n (%)** | 3 (2·4) | 13 (10·2) | 36 (28·3) | 20 (15·7) | 51 (40·2) | 4 (6·1) | 5 (7·6) | 12 (18·2) | 8 (12·1) | 29 (43·9) | 50 (42·0) | 42 (35·3) | 13 (10·9) | 33 (27·7) | 6 (5·0) | 19 (32·2) | 18 (30·5) | 12 (20·3) | 20 (33·9) | 8 (13·6) |
| **>300 to <1000, n (%)** | 43 (33·9) | 56 (44·1) | 51 (40·2) | 54 (42·5) | 23 (18·1) | 13 (19·7) | 23 (34·8) | 29 (43·9) | 24 (36·4) | 14 (21·2) | 28 (23·5) | 29 (24·4) | 2 (1·7) | 21 (17·6) | 9 (7·6) | 21 (35·6) | 14 (23·7) | 7 (11·9) | 11 (18·6) | 7 (11·9) |
| **≥1000, n (%)** | 81 (63·8) | 57 (44·9) | 20 (15·7) | 48 (37·8) | 8 (6·3) | 49 (74·2) | 35 (53·0) | 17 (25·8) | 30 (45·5) | 8 (12·1) | 8 (6·7) | 6 (5·0) | 4 (3·4) | 7 (5·9) | 2 (1·7) | 9 (15·3) | 11 (18·6) | 2 (3·4) | 11 (18·6) | 2 (3·4) |

* Day 152 post-BNT162b2 in the control group.

## Table S9. Summary statistics for cellular immune response related to BNT162b2 administration time (day 0)

|  | **N** | **Interventional group,**  **geometric mean [95% CI]** | **N** | **Control group,**  **geometric mean [95% CI]** | **Ratio [95% CI]** | **p-value** |
| --- | --- | --- | --- | --- | --- | --- |
| IFN-γ day -28 | - | - | 53 | 151·63 [114·09;201·53] | - | - |
| IFN-γ day -14 | - | - | 53 | 122·67 [88·55;169·95] | - | - |
| IFN-γ day 0 | 102 | 129·63 [103·51;162·35] | 33 | 173·67[118·89;253·69] | 0·75 [0·48;1·17] | 0·1976 |
| IFN-γ day 14 | 98 | 521·22 [422·44;643·09] | - | NA | - | - |
| IFN-γ day 28 | 79 | 380·93 [309·07;469·5] | 30 | 485·32 [343·51;685·68] | 0·78 [0·53;1·17] | 0·2279 |
| IFN-γ day 90 | 53 | 252·14 [191·21;332·47] | - | NA | - | - |
| IFN-γ day 180* | 51 | 223·8 [166·25;301·28] | 34 | 171·23[120·15;244·02] | 1·31[0·83;2·07] | 0·2485 |
| IL-2 day -28 | - | - | 53 | 143·89 [114·98;180·07] | - | - |
| IL-2 day -14 | - | - | 53 | 70·45 [46·39;107] | - | - |
| IL-2 day 0 | 102 | 129·73 [108·5;155·12] | 35 | 162·22 [124·77;210·91] | 0·8 [0·57;1·12] | 0·1948 |
| IL-2 day 14 | 98 | 132·72 [92·78;189·86] | - | NA |  |  |
| IL-2 day 28 | 79 | 244·07 [204·89;290·74] | 28 | 299·2 [217·81;411·01] | 0·82 [0·58;1·15] | 0·2445 |
| IL-2 day 90 | 52 | 190·28 [150·18;241·08] | - | NA | - | - |
| IL-2 day 180* | 51 | 171·54 [133·08;221·12] | 34 | 170·25 [122·24;237·11] | 1·01 [0·67;1·51] | 0·9706 |

* Corresponded to day 152 post-dose in the control group. NA: not available (titres at days 7 and 14 post-BNT162b2 not planned in the control group; titres at day 90 post-dose not planned in the control group)

## Figure S4. Plot of Total Sample Size versus Power for Two-Sample t-test for Mean Ratio


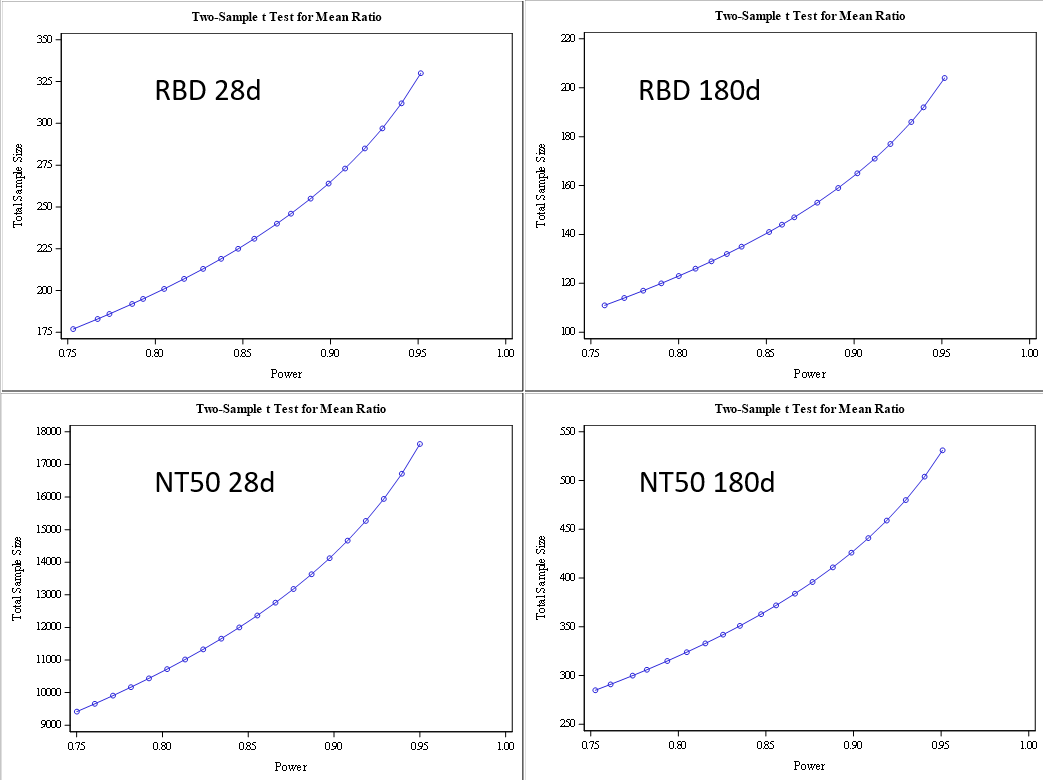


The analysis above shows that sample size was appropriate to evaluate differences between mean log10 RBD28d ratio equal to 0.77 and coefficient of variation equal to 0.68, for mean log10 RBD180d mean ratio equal to 0.62 and coefficient of variation equal to 1.09, and between mean log10 NT50 equal to 0.67 and coefficient of variation equal to 1.8. Sample size for NT5028d mean ratio and coefficient of variation equal to 0.95 and 1.10, respectively, would have need to be higher than actual sample size; however we do not consider this small difference clinically relevant to justify a larger sample size

**Table S10. Baseline characteristics of the groups under comparisons at day 180**

|  | **Interventional group (n=416)** | **Control group**  **(n= 198)** | **Overall**  **(n=614)** |
| --- | --- | --- | --- |
| **Sex** |  |  |  |
| Male | 175 (42 1%) | 84 (42 4%) | 259 (42 2%) |
| Female | 241 (57 9%) | 114 (57 6%) | 355 (57 8%) |
| **Age (years)** | 44·20 (8·66) | 44·68 (8·53) | 44 36 (8 61) |
| **Age group** |  |  |  |
| 18-49 years | 270 (64 9%) | 121 (61 1%) | 391 (63 7) |
| 50-59 years | 146 (35 1%) | 77 (38 9%) | 223 (36 3) |
| **Days between vaccines** | 61 30 (5 63) | 88 93 (5 92) | 70 21 (14 14) |

Data are n (%) and mean (SD).

## Table S11. Effect coefficient of interventional group versus control group adjusted by sex, age, and time between vaccine doses (model 1) and adjusted by sex, age, time between vaccine doses, comorbidities and bmi (model 2).

| **Variable** | **Interventional group versus control group coefficient– model 1** | **Interventional group versus control group coefficient– model 2** |
| --- | --- | --- |
| RBD day 180 post-BNT162b2* | -0.1760 (-0.2277; -0.1242) | -0.1747 (-0.2265; -0.1230) |
| NT50 (G614) day 180 post-BNT162b2* | -0.1250 (-0.2515; 0.0007) | -0.1285 (-0.2550; -0.0020) |
| NT50 (Alpha) day 180 post-BNT162b2* | -0.2116 (-0.3396; -0.0836) | -0.2205 (-0.3482; -0.0928) |
| NT50 (Beta) day 180 post-BNT162b2* | -0.1514 (-0.2823; -0.0205) | -0.1558 (-0.2869; -0.0248) |
| NT50 (Delta) day 180 post-BNT162b2* | -0.2300 (-0.3879; -0.0721) | -0.2407 (-0.3980; -0.0833) |
| NT50 (Omicron) day 28 post-BNT162b2* | -0.1404 (-0.2619; -0.0189) | -0.1451 (-0.2670; -0.0233) |
| IFN day 180 post-BNT162b2* | 0.0343 (-0.1145; 0.1832) | 0.0346 (-0.1139; 0.1830) |
| IL2 day 180 post-BNT162b2* | -0.0143 (-0.1444; 0.1159) | -0.0144 (-0.1442; 0.1153) |

The table S11 above show the beta coefficient in the regression model by treatment (interventional group versus control group) for outcome variables to 180 days: Humoral immunogenicity, measured by immunoassay for SARS-CoV-2 receptor binding domain (RBD), antibody functionality using pseudovirus neutralisation assays for the reference (G614), Alpha, Beta, Delta, and Omicron variants, as well as cellular immune response using interferon-γ and IL-2 immunoassays. The model 1 was adjusted by sex, age, and time between vaccine doses and model 2 was adjusted by sex, age, time between vaccine doses, comorbidities and BMI. The models incorporated the raw data response with distribution lognormal data, and for humoral immunogenicity a truncated regression model was used, as specified in section Statistical Analysis of the manuscript. Similar results was found between model 1 and model 2.

# Supplementary Methods

## Table S12. Number of subjects with full data for each variable analysed

| **Variable** | **Interventional group**  **(n=444)** | **Control group**  **(n=223)** |
| --- | --- | --- |
| RBD day 0 | 444 | 219 |
| RBD day 28 post-BNT162b2 | 439 | 212 |
| RBD day 90 post-BNT162b2 | 425 | NA |
| RBD day 180 post-BNT162b2* | 416 | 198 |
| NT50 day 0 | 129 | 69 |
| NT50 day 28 post-BNT162b2 | 127 | 66 |
| NT50 (G614) day 28 post-BNT162b2 | 127 | 66 |
| NT50 (Alpha) day 28 post-BNT162b2 | 127 | 66 |
| NT50 (Beta) day 28 post-BNT162b2 | 127 | 66 |
| NT50 (Delta) day 28 post-BNT162b2 | 127 | 66 |
| NT50 (Omicron) day 28 post-BNT162b2 | 127 | 66 |
| NT50 day 90 post-BNT162b2 | 127 | NA |
| NT50 day 180 post-BNT162b2* | 119 | 59 |
| NT50 (G614) day 180 post-BNT162b2 | 119 | 59 |
| NT50 (Alpha) day 180 post-BNT162b2* | 119 | 59 |
| NT50 (Beta) day 180 post-BNT162b2* | 119 | 59 |
| NT50 (Delta) day 180 post-BNT162b2* | 119 | 59 |
| NT50 (Omicron) day 28 post-BNT162b2* | 119 | 59 |
| IFN day 0 | 102 | 53 |
| IFN day 28 post-BNT162b2 | 79 | 30 |
| IFN day 90 post-BNT162b2 | 53 | NA |
| IFN day 180 post-BNT162b2* | 51 | 34 |
| IL2 day 0 | 102 | 53 |
| IL2 day 28 post-BNT162b2 | 79 | 28 |
| IL2 day 90 post-BNT162b2 | 52 | NA |
| IL2 day 180 post-BNT162b2* | 51 | 34 |

* Corresponded to day 152 post-dose in the control group. NA: not available (variables at day 90 post-dose not planned in the control group)

## Pseudo-virus neutralisation assays

*Cell lines*

The Vero E6 (African green monkey kidney) cell line was kindly provided by Dr. A. Alcami (CBM Severo Ochoa, Madrid). HEK-293T (National Institute for Biological Standards and Control (NIBSC]) and Vero E6 cells were cultured in DMEM supplemented with 10% FCS, 2 mM L glutamine and 100 units/ml penicillin and streptomycin (Lonza).

*Pseudo-virus generation and neutralisation assay*

The codon-optimized sequence from Ou et al. (1) was modified by synthesis (GeneArt Gene Synthesis, ThermoFisher Scientific) to generate the different spikes of SARS-CoV-2 virus: D614G (as the reference variant), Alpha (∆69-70, ∆144, N501Y, A570D, D614G, P681H, T716I, S982A, D1118H), Beta (L18F, D80A, D215G, ∆242-244, K417N, E484K, N501Y, D614G, A701V), Delta (T19R, ∆156-157, R158G, L452R, T478K, D614G, P681R, D950N), and Omicron (A67V, ∆69-70, T95I, G142D, ∆143-145, N211I, ∆212-212, G339D, S371L, S373P, S375F, S477N, T478K, E484A, Q493R, G496S, Q498R, N501Y, Y505H, T547K, D614G, H655Y, N679K, P681H, D796Y, N856K, Q954H, N969K, L981F). All these sequences were synthesized with a deletion in the last 19 amino acids from the original spike and were inserted in pcDNA3.1D/V5-His-TOPO between BamHI and XbaI sites. The pcDNA-VSV-G plasmid contains the cDNA encoding the vesicular stomatitis virus G protein and was obtained from Dr. Arenzana-Seisdedos (Institute Pasteur, Paris, France).

NL4.3 pseudotypes were generated with the previously described plasmid pNL4-3ΔenvRen (2). Briefly, Renilla luciferase reporter pseudovirus were prepared by co-transfecting HEK-293T cells with pNL4-3ΔenvRen backbone and viral envelope protein expression plasmid pcDNA3.1-S-CoV2Δ19 or pcDNA-VSV-G using the calcium phosphate method. The medium was changed 18 hours after transfection, and 48 hours post-transfection cell culture supernatants were harvested, clarified by centrifugation at 500 x g for 5 min, and frozen at -80°C. The amount of p24 antigen in the supernatants was quantified by electrochemiluminescence Immunoassay (Roche Diagnostic).

To measure neutralisation activity of plasma from donors, four-fold serial dilutions of heat-inactivated sera (1:32-1:131072) were preincubated with titrated pseudoviruses (10 ng of p24 Gag/well) for 1 hour at 37 °C. Thereafter, 100 μl of the mixture was added to Vero E6 cells plated the previous day at 5 x 103 cells/well in 100 μl medium in 96-well plates. The culture medium was refreshed after 16 hours. At 48 h post-infection, cells were lysed, and viral infectivity was assessed by measuring luciferase activity (Renilla Luciferase Assay, Promega, Madison, WI, USA) using a 96-well plate luminometer LB 960 Centro XS³ (Berthold Technologies, Oak Ridge,TN, USA). The titres of neutralising antibodies were calculated as 50% inhibitory dose (neutralising titer 50, NT50), expressed as the highest dilution of plasma which resulted in a 50% reduction of luciferase activity compared to control without plasma. Sigmoid curves were generated and NT50s were calculated by non-linear regression using GraphPad Prism version 9.3.1 (GraphPad Software, Inc.). VSV-G pseudoviruses were used as a control of specificity in neutralisation testing.

1. Ou X, Liu Y, Lei X, Li P, Mi D, Ren L, Guo L, Guo R, Chen T, Hu J, Xiang Z, Mu Z, Chen X, Chen J, Hu K, Jin Q, Wang J, Qian Z. Characterization of spike glycoprotein of SARS-CoV-2 on virus entry and its immune cross-reactivity with SARS-CoV. Nat Commun. 2020;11: 1620.

2. Garcia-Perez J, Sanchez-Palomino S, Perez-Olmeda M, Fernandez B, Alcami J. A new strategy based on recombinant viruses as a tool for assessing drug susceptibility of human immunodeficiency virus type 1. J Med Virol. 2007 Feb;79(2):127-37.

## Cellular immunity quantification

*SARS-CoV-2 Peptide pools*

SARS-CoV-2 peptide pools of Prot S/S1/S+ from Miltenyi, were used as recently reported (1).

*Whole blood culture with SARS-CoV-2 peptide pools*

320 μl of whole blood drawn on the same day were mixed with 80 μl RPMI and stimulated with pools of SARS-CoV-2 peptides (S; 2 μg/ml) or DMSO control. After 15 hours of culture, the supernatant (plasma) was collected and stored at -80ºC until quantification of cytokines as recently described (2).

*Cytokine quantification and analysis*

Cytokine concentrations in the plasma were quantified using Ella with microfluidic multiplex cartridges measuring IFN-γ and IL-2 following the manufacturer’s instructions (ProteinSimple, San Jose, CA, USA). The level of cytokines present in the plasma of DMSO controls was subtracted from the corresponding peptide pool stimulated samples.

1. Lozano-Ojalvo D, Camara C, Lopez-Granados E, Nozal P, Del Pino-Molina L, Bravo-Gallego LY, Paz-Artal E, Pion M, Correa-Rocha R, Ortiz A, Lopez-Hoyos M, Iribarren ME, Portoles J, Rojo-Portoles MP, Ojeda G, Cervera I, Gonzalez-Perez M, Bodega-Mayor I, Montes-Casado M, Portoles P, Perez-Olmeda M, Oteo J, Sanchez-Tarjuelo R, Pothula V, Schwarz M, Brahmachary M, Tan AT, Le Bert N, Berin C, Bertoletti A, Guccione E, Ochando J. Differential effects of the second SARS-CoV-2 mRNA vaccine dose on T cell immunity in naive and COVID-19 recovered individuals. Cell Rep. 2021 Aug 24;36(8):109570.

2. Le Bert N, Clapham HE, Tan AT, Chia WN, Tham CYL, Lim JM, et al. Highly functional virus-specific cellular immune response in asymptomatic SARS-CoV-2 infection. J Exp Med. 2021;218(5).

# Supplementary Author List

## CombiVacS Vaccine Trial Group

| AFFILIATION | NAME | SURNAME |
| --- | --- | --- |
| **La Paz University Hospital, Madrid** | Lucía  Amelia  Lucía  Elena  Enrique  Stefan Mark  Alicia  Irene  Mikel  Jaime  Paula  Marta  Cristina  Vega  Raquel  Laura  Julio  Antonio  Eduardo  Carmen  Esther  Pilar  María  Victoria  Paloma  Amparo  Rocío  Silvia  Cristina  Marina  Fernando  Marta  Blanca | Martínez de Soto  Rodríguez Mariblanca  Díaz García  Ramírez García  Seco Meseguer  Stewart Balbás  Marín Candón  García García  Urroz Elizalde  Monserrat Villatoro  de la Rosa  Sanz García  López Crespo  Mauleón Martínez  de Madariaga Castell  Vitón Vara  García Rodríguez  Buño  López Granados  Cámara  Rey Cuevas  Ayllon García  Jiménez González  Hernández Rubio  Moraga Alapont  Sánchez  Prieto  Llorente Gómez  Miragall Roig  Aparicio Marlasca  de la Calle  Arsuaga  Duque |
| **Cruces University Hospital, Biocruces, Bizkaia** | Susana  Aitor  Ana  Mikel  Dolores  Ana Belén  Gustavo  Olaia  Josu  Maria Angeles  Alejandro  Inés  Rosa  Begoña  Laura  Sara | Meijide  García de Vicuña  San Expósito  Gallego  García-Vázquez  de la Hoz  Pérez-Nanclares  Velasco  Aurrekoetxea  Lázaro  García Castaño  Urrutia  Martínez  Calvo  Saso  Gómez |
| **National Centre of Microbiology, Institute of Health Carlos III – ISCIII, Madrid (Spain)** | Isabel  Concepción  Giovanni  Javier  María Jesús  Francisco  Esther  Almudena  Paloma  Jana  Isabel  Marcos  Pilar | Jado  Perea  Fedele  Hernández  Llamas  Díez-Fuertes  Calonge  Cascajero  Jiménez-Santana  Baranda  Cervera  Berges-Buxeda  Portolés |
| **Clinic Hospital, Barcelona (Spain)** | Marta  Lourdes E  Mª Ángeles  Laura  Sulayman  Sara  Montserrat  Sebastiana  Anna  Anna  Victoria  Antoni  Begoña  Elisenda  Sheila  Francisco Javier  Cristina  Laura  Juan José  Patricia  Josep Lluís  Jordi | Aldea  Barón-Mira  Marcos  Granés  Lazaar  Herranz  Malet  Quesada  Vilella  Llupià  Olivé  Trilla  Gómez  González  Romero  Gámez  Casals  Burunat  Castelló  Fernández  Bedini  Vila |
| **Vall d’Hebron University Hospital, Barcelona (Spain)** | Xavier  Susana  Blanca  Oleguer  Cesar  José Angel  Lluis  Sonia  Judith  José  Carla  Lina  Aitana  Carla  Laia  Gisela  Carmen  Gloria  Esther  Margarita  Ana  Julia  Elena Ballarin  Eulàlia  Lourdes | Martínez-Gómez  Otero-Romero  Borras-Bermejo  Parés-Badell  Llorente  Rodrigo-Pendás  Armadans  Uriona  Riera  Santos  Sans  Camacho  Plaza  Aguilar  Pinos  Gili  Altadill  Torres  Palacio  Torrens  Feliu  Calonge  Ballarin Alins  Pérez-Esquirol  Vendrell Bosch |
| **Hospital Clínico San Carlos – IDISSC, Madrid (Spain)** | Leonor  Manuel  Jose Antonio  Esperanza  Natalia  Ana Belén  Teresa  Ouhao  Angel  Daniel  Verónica  Oliver  Natalia  María Aránzazu  Carmen  Agustín | Laredo  Sanchez-Craviotto  Gil-Marin  Gonzalez-Rojano  Rodriguez-Galán  Rivas-Paterna  Iglesias  Zhu-Huang  Hernández-Bartolomé  Lozano-Martín  Alvarez-Morales  Astasio  Pérez-Macías  Urrutia-de-la-Plaza  Sanz  Molina |

## Data and Safety Monitoring Board (DSMB)

| **Prof. Oliver A. Cornely, MD, FACP, FIDSA, FAAM, FECMM** | 1. University of Cologne, Faculty of Medicine and University Hospital Cologne, Department I of Internal Medicine, Excellence Center for Medical Mycology (ECMM), Cologne, Germany  2. University of Cologne, Faculty of Medicine and University Hospital Cologne, Chair Translational Research, Cologne Excellence Cluster on Cellular Stress Responses in Aging-Associated Diseases (CECAD), Cologne, Germany  3. University of Cologne, Faculty of Medicine and University Hospital Cologne, Clinical Trials Centre Cologne (ZKS Köln), Cologne, Germany  4. University of Cologne, Faculty of Medicine and University Hospital Cologne, Center for Molecular Medicine Cologne (CMMC), Cologne, Germany  5. German Centre for Infection Research (DZIF), Partner Site Bonn-Cologne, Cologne, Germany |
| --- | --- |
| **Prof. Jesús Rodríguez Baño, MD, PhD** | 1. Servicio de Enfermedades Infecciosas, Hospital Universitario Virgen Macarena, Sevilla, Spain  2. Departamento de Medicina, Universidad de Sevilla, Sevilla, Spain  3. Instituto de Biomedicina de Sevilla, Sevilla, Spain  4. Red Española de Investigación en Patología Infecciosa (REIPI-ISCIII) |
| **Dr. Clara Menéndez, MD, PhD** | 1. Maternal, Child and Reproductive Health Initiative. Servicio de Salud Internacional ISGlobal. Barcelona Institute for Global Health, Barcelona, Spain  2 Hospital Clinic, Universitat de Barcelona, Barcelona, Spain |

## Steering Committee (SC)

| **Name** | **Role** | **Affiliation** |
| --- | --- | --- |
| **Cristobal Belda Iniesta, MD, PhD** | Study Chair | Instituto de Salud Carlos III. Madrid, Spain |
| **Jesús Frías Iniesta, MD, PhD** | Investigator Coordinator | Clinical Pharmacology Department. La Paz University Hospital. Spanish Clinical Research Network – SCReN- ISCIII. Madrid, Spain |
| **Jose R Arribas, MD, PhD** | Clinical Director | Infectious Diseases Unit. Internal Medicine Service. La Paz University Hospital. Madrid, Spain |
| **Antonio J. Carcas, MD, PhD** | Scientific Coordinator | Clinical Pharmacology Department. La Paz University Hospital. Spanish Clinical Research Network – SCReN-ISCIII. Madrid, Spain |
| **Alberto M. Borobia, MD, PhD** | Principal Investigator. Management Coordinator | Clinical Pharmacology Department. La Paz University Hospital. Spanish Clinical Research Network – SCReN-ISCIII. Madrid, Spain |
| **Luis Castaño, MD, PhD** | Principal Investigator | Cruces University Hospital, Biocruces Bizkaia HRI, UPV/EHU, OSAKIDETZA. Barakaldo, Spain  CIBERDEM, CIBERER. ISCIII. Madrid, Spain  Endo-ERN. Leiden, The Netherlands  Spanish Clinical Research Network – SCReN-ISCIII. Madrid, Spain |
| **M. Jesús Bertran, PhD** | Principal Investigator | Preventive Medicine and Epidemiology Department. Hospital Clínic de Barcelona. Barcelona, Spain |
| **Magdalena Campins, MD, PhD** | Principal Investigator | Preventive Medicine and Epidemiology Department. Vall d’Hebron University Hospital. Barcelona, Spain |
| **Antonio Portolés, MD, PhD** | Principal Investigator | Clinical Pharmacology Department. Hospital Clínico San Carlos - IdISSC; Pharmacology&Toxicology Dpt., UCM; Spanish Clinical Research Network – SCReN-ISCIII. Madrid, Spain |
| **Inmaculada Fuentes, MD, PhD** | CTU Manager | Clinical Research Support Unit. Vall d'Hebron Research Institut (VHIR). Clinical Pharmacology Service. Vall d’Hebron University Hospital. Barcelona, Spain  Spanish Clinical Research Network – SCReN- ISCIII. Madrid, Spain |
| **Joan A. Arnaiz** | CTU Manager | Spanish Clinical Research Network – SCReN-ISCIII. Madrid, Spain |
| **Jose Alcamí, MD, PhD** | Principal Investigator | AIDS Immunopathogenesis Unit. Centro Nacional de Microbiologia. Instituto de Salud Carlos III. Madrid, Spain |
| **Jordi Cano Ochando, PhD** | Principal Investigator | Centro Nacional de Microbiologia. Instituto de Salud Carlos III. Madrid, Spain |
| **Mayte Pérez Olmeda, PhD** | Principal Investigator | Serology laboratory. Centro Nacional de Microbiologia. Instituto de Salud Carlos III. Madrid, Spain |
| **Jon Salmatón García, MD, PhD** | External member | University of Cologne, Faculty of Medicine and University Hospital Cologne, Department I of Internal Medicine, Excellence Center for Medical Mycology (ECMM). Cologne, Germany  University of Cologne, Faculty of Medicine and University Hospital Cologne, Chair Translational Research, Cologne Excellence Cluster on Cellular Stress Responses in Aging-Associated Diseases (CECAD). Cologne, Germany |
| **Anxo Fernández-Ferreiro, PhD** | External member | Clinical Pharmacology Group & Pharmacy Department, University Clinical Hospital-Health Research Institute of Santiago de Compostela (IDIS-SERGAS). Santiago de Compostela, Spain |
| **Ferrán Torres, MD, PhD** | External member | Medical Statistics core facility, Clinical Pharmacology Department, Hospital Clinic Barcelona. Barcelona, Spain  Biostatistics Unit, Faculty of Medicine, Universitat Autònoma de Barcelona. Barcelona, Spain |
| **Magi Farré, MD, PhD** | External member | Clinical Pharmacology Department. Gernans Trias i Pujol University Hospital. Universitat Autònoma de Barcelona. Barcelona, Spain  Spanish Clinical Research Network – SCReN- ISCIII. Madrid, Spain |

# Study Protocol


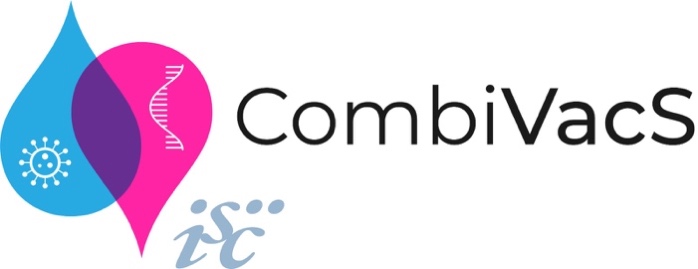


**Protocol Title:**

**A Phase 2, Comparative, Randomised, Adaptive Trial to Evaluate the safety and immunogenicity of one dose of COMIRNATY® in subjects that had received one dose of VAXZEVRIA®**

**Protocol Version and Date:** 1.2 / May 21, 2021

(Includes non-substantial modification number 2)

**Protocol Code:** CombiVacS

**Brief Title:** Vaccination with COMIRNATY in subjects with a VAXZEVRIA first dose.

CombiVacS is a phase 2 randomized, adaptive trial to evaluate the immunogenicity and safety of one dose of mRNA COVID19 vaccine (COMIRNATY – Pfizer) in subjects having received a single previous dose of a viral vector COVID19 vaccine (VAXZEVRIA – AstraZeneca)

**Study Phase:** 2

**Sponsor Name:**

Instituto de Salud Carlos III (ISCIII)

**Regulatory Agency Identifier Number:**

| EudraCT | 2021-001978-37 |
| --- | --- |

**Approval Date:**

Sponsor Signatory:

|  |  | **Date** |
| --- | --- | --- |

# Protocol Summary

## Synopsis

| **Name of Sponsor** | **Instituto de Salud Carlos III (ISCIII)** |
| --- | --- |
| **Protocol Number/Title:** | A Phase 2, Comparative, Randomised, Adaptive Trial to Evaluate the safety and immunogenicity of one dose of COMIRNATY in subjects that had received one dose of VAXZEVRIA |
| **Indication:** | Covid-19 prevention |
| **Hypothesis** | Immunogenicity in patients who have already received one dose of VAXZEVRIA will be higher when it is followed by one dose of COMINARTY administered 8-12 weeks later. |
| **Trial Objectives:** | **Primary Immunogenicity Objective:**   - To assess the humoral immune response against SARS-CoV-2, 14 days after a vaccination with COMIRNATY in subjects that had received a previous single dose of VAXZEVRIA (Group 1) as compared with no dosing (Group 2).   **Secondary Immunogenicity Objectives:**   - To assess the humoral immune response against SARS-CoV-2 28 days after a vaccination with COMIRNATY in subjects that received a previous single dose of VAXZEVRIA . - To assess the long-term (up to 1 year) humoral immune response against SARS-CoV-2 of a dose of COMIRNATY in subjects that received a previous single dose of VAXZEVRIA. - To assess the humoral immune response against viral variants of SARS-CoV-2, 14 and 28 days after a dose of COMIRNATY in subjects that received a previous single dose of VAXZEVRIA.   **Exploratory objective:**   - To evaluate the relationship between the immune response measured as NAV (Neutralizing antibodies) and antibodies against SARS-CoV-2 spike protein measured by immunoassay.   **Secondary efficacy objectives:**   - To assess the occurrence of symptomatic molecularly confirmed COVID-19 and severity of COVID-19 signs and symptoms after the administration of a dose of COMIRNATY in subjects that received a prior single dose of VAXZEVRIA.   **Primary Safety Objectives:**   - To evaluate the safety of a dose of COMIRNATY in subjects that received a previous single dose of VAXZEVRIA. |
| **Trial Design Overview:** | This is a randomized, non-blinded, controlled, adaptive, multicenter, Phase II study in subjects aged ≥18 years and in good health or stable clinical situation that have received a previous single dose of VAXZEVRIA.  This is a phase 2 adaptive trial developed to evaluate the immunogenicity of a dose of COMIRNATY after a previous single dose of VAXZEVRIA. A stratification will be made based on the following factors: study site, sex and age. This protocol allows to test the immunogenicity and safety of a heterologous vaccination strategy after a previous single dose of VAXZEVRIA.  Subjects will be randomized to immediately receive or not a dose of COMIRNATY in a ratio of 2:1.  If primary analysis at 14 days confirms the starting hypothesis, subjects randomized to no vaccination will be considered for administration of one dose of COMINARTY at day 28^th^ according to Public Health Department of the Ministry of Health recommendations on heterologous vaccination. If vaccinated, subjects will be followed at the time points defined in the flowchart.  In case the primary analysis does not confirm the starting hypothesis, subjects will be followed at the time points defined in the flow-chart without administration of COMIRNATY.  In case Public Health Department gave instructions to give the second dose of VAXZEVRIA to those subjects having received the first dose, subject will be followed as indicated in flowchart of Group 2.  Other heterologous vaccination strategies could be incorporated if deemed necessary for public health reasons. This could include the use of different vaccination strategies including those already marketed vaccines for comparative assessment of their safety and efficacy on SARS-CoV-2 and its variants.  The different intervention arms planned (and its corresponding control arm) can be carried-out in different trial sites, thus not all trial sites must participate in all arms. See trial schematic for more details. |
| **Intervention and control arms** | Intervention arm (Group 1): administration of a COMINARTY dose.  Control arm (Groups 2): no administration of COMINARTY dose. |
| **Randomization and blinding** | **Randomization**.  A central randomization stratified by study site and age will be used.  Randomization will be in a ratio of 2:1  **Blinding.**  No blinding is planned in this trial |
| **Participant Population:** | **Inclusion criteria**   1. Adult subjects (18 years old) having received prior VAXZEVRIA vaccination between 8 and 12 weeks before the screening visit. 2. Participants must provide consent indicating that he or she understands the purpose, procedures and potential risks and benefits of the study, and is willing to participate in the study. 3. Subjects in good health or stable clinical situation. 4. Participant is willing and able to adhere to the procedures specified in this protocol.   **Exclusion criteria**   1. Participant has a clinically significant acute illness (this does not include minor illnesses such as diarrhea or mild upper respiratory tract infection) or temperature ≥38.0ºC within 24 hours prior to the planned dose of study vaccine. 2. Participant has a known or suspected allergy or history of anaphylaxis or other serious adverse reactions to COMIRNATY excipients. 3. Subjects with any contraindication to the administration of COMIRNATY, included pregnancy. 4. Subjects with prior documented COVID19 since VAXZEVRIA vaccination. 5. Subjects have symptoms or signs compatible with COVID19. 6. Subjects participating in a clinical trial in the last three months. 7. Any condition or situation precluding or interfering the compliance with the protocol.   **Number of participants:**  The total planned number of patients to be included is 600 (400 in COMINARTY arm and 200 in no vaccination arm). |
| **Medicinal Product, Dose and Mode of Administration:** | 1) COMIRNATY at standard dose will be administered at the standard dose defined in the summary of product characteristics in subjects randomized to receive the dose immediately or in control arm if primary analysis at 14 days confirms the starting hypothesis and no recommendations against are issued by the Public Health Department. |
| **Primary immunogenicity endpoint:**   - Serological response to vaccination (antibodies against SARS-CoV-2 spike protein) as measured by immunoassay, 14 days after vaccine dose (Group 1) or randomization (Group 2) .   **Secondary immunogenicity endpoint:**   - Serological response to vaccination (antibodies against SARS-CoV-2 spike protein) as measured by immunoassay, 28 days after vaccine dose (Group 1) or randomization (Group 2) . - SARS-CoV-2 neutralization as measured by virus neutralization assay, 14 and 28 days after vaccine dose (Group 1) or randomization(Group 2) . - Serological response to vaccination (antibodies against SARS-CoV-2 spike protein) as measured by immunoassay at 3, 6 and 12months after randomization. - Neutralizing antibody titers at 3, 6 and 12 months after randomization.   **Secondary efficacy endpoints:**   - The number of participants with molecularly confirmed COVID-19. - Presence and severity of COVID-19 signs and symptoms as measured by Symptoms of Infection with Coronavirus-19 (SIC).   **Primary safety endpoints:**   - Solicited local and systemic adverse events (AEs) for a period of 7 days after vaccination. - Unsolicited local and systemic adverse events (AEs) for a period of 28 days after vaccination - Serious adverse events (SAEs) throughout the study (from randomization until end of the study). - Medically-attended adverse events (MAAEs) from the day of vaccination until 6 months after the last vaccination. - Abnormal laboratory parameters at 1 month visit after COMIRNATY vaccination. | |
| **Statistical Analysis.**  **Primary immunogenicity endpoint** is the titer of antibodies binding to the SARS-CoV-2 S protein on14^th^ day measured by immunoassay.  Secondary immunogenicity endpoint is NAV (FRNT50 GMT, which represent the reciprocal dilution of serum that neutralizes 50% of the input virus expressed as the geometric men) evaluated in the randomization visit (previously COMIRNATY vaccination) and on 14, 28 days and 3, 6 and 12 months after dosing.  **Primary statistical analysis** will be performed on day 14^th^ after randomization.  **Main comparisons are**:   - Immune response measured by immunoassay on 14 days after the randomization (±2 days).   Other comparisons will include:   - Immune response (antibodies against SARS-CoV-2 spike protein) measured by immunoassay at 7 days after the randomization (±2 days). - Immune response (antibodies against SARS-CoV-2 spike protein) measured by immunoassay at 28 days after the randomization (±2 days) - Immune response measured by NAV at 14 days after the randomization (±2 days). - Immune response measured by NAV at 28 days after the randomization (±2 days) - Correlation of immune response measured by NAV and immunoassay (antibodies against SARS-CoV-2 spike protein). - Change in immune response from baseline (randomization visit) to 14 and 28 days after COMIRNATY vaccination in the immediate and control groups. - Differences in immune response at the different time points by both immunoassay (antibodies against SARS-CoV-2 spike protein) and NAV tests. - Percentage of patients reaching a FRNT50 GMTs >1000 at 14 and 28 days (or the figure having best evidence in the literature at the time of analysis) - Maintaining a FRNT50 GMTs >1000 (or the figure having best evidence in the literature at the time of analysis) at 3, 6 and 12 months.   A full description of statistical analysis will be made in the SAP.  **Safety Analysis:**  All clinical safety data will be listed by participant, time form vaccination dose and treatment arm. Continuous variables will be summarized using sample size (N), mean, standard deviation, median, minimum, and maximum. Frequency counts will be reported for categorical data.  A Data and Safety Monitoring Committee (DSMC) will review data for decisions on arms incorporation and withdrawal and will ensure that the appropriate oversight and monitoring in conducting the clinical trial. Interim analysis could be planned as necessary in each sub-protocol. | |
| **Trial Duration:**  First analysis (completed 14 day) will be available about 5 weeks after first patient inclusion.  Each patient will be followed for 1 year.  **End of Study Definition:**  The end of study is considered as the last visit for the last participant in the study | |

## Flowchart.

**Group 1**

| **Visit number** |  | **V1** | **V2** | **V3** | **V4** | **V5** | **V6** | **V7** |  |  |  |  |  |
| --- | --- | --- | --- | --- | --- | --- | --- | --- | --- | --- | --- | --- | --- |
| **Procedure** | **Screening** | **Randomization/ Boost** | **Visit 2** | **Visit 3** | **Visit 4** | **Visit 5** | **Visit 6** | **End of study** |  |  |  |  |  |
| **Day ± window** | 0 to 7 days before boost vaccination | 0 | 7±2 | 14 ±3 | 28±3 | 90±10 | 180±10 | 360±14 |  |  |  |  |  |
| **Screening for eligibility** |  |  |  |  |  |  |  |  |  |  |  |  |  |
| Eligibility check | X |  |  |  |  |  |  |  |  |  |  |  |  |
| Informed consent | X |  |  |  |  |  |  |  |  |  |  |  |  |
| PCR |  | X |  |  |  |  |  |  |  |  |  |  |  |
| **Baseline procedures** |  |  |  |  |  |  |  |  |  |  |  |  |  |
| Randomization |  | X |  |  |  |  |  |  |  |  |  |  |  |
| Record demographic information | X |  |  |  |  |  |  |  |  |  |  |  |  |
| Pregnancy test in urine | X |  |  |  |  |  |  |  |  |  |  |  |  |
| Record medical history | X |  |  |  |  |  |  |  |  |  |  |  |  |
| Record medication history | X | X | | | | | | X |  |  |  |  |  |
| Complete physical examination | X |  |  |  |  |  |  |  |  |  |  |  |  |
| Measure vital signs^1^ | X |  |  | X | X |  |  |  |  |  |  |  |  |
| Lab determinations | X^2^ | X^2^ |  |  | X |  |  |  |  |  |  |  |  |
| Vaccination |  | X |  |  |  |  |  |  |  |  |  |  |  |
| **Endpoint assessment** |  |  |  |  |  |  |  |  |  |  |  |  |  |
| Assess acute reactions for at least 15 minutes after study intervention administration |  | X |  |  |  |  |  |  |  |  |  |  |  |
| Solicited local and systemic adverse events (AEs) for 7 days after vaccine . Via E-diary |  | X | |  |  |  |  |  |  |  |  |  |  |
| Unsolicited local and systemic adverse events (AEs) for 28 days after vaccine . Via E-diary |  | X^3^ | | | |  |  |  |  |  |  |  |  |
| Unsolicited SAEs |  | X | | | | | | |  |  |  |  |  |
| Medically-attended adverse events (MAAEs) |  | X | | | | | |  |  |  |  |  |  |
| Immune response evaluation |  | X | X | X | X | X | X | X |  |  |  |  |  |

1. Temperature, blood pressure and heart rate

2. Lab determination could be performed either at screening vist or randomization3. Two days per week

**Group 2 (if crossing over is decided) ***

| **Visit number** |  | **V1** | **V2** | **V3** | **V4** | **V5** | **V6** | **V7** |
| --- | --- | --- | --- | --- | --- | --- | --- | --- |
| **Procedure** | **Screening** | **Randomization** | **Visit 2** | **Visit 3** | **Visit 4 – Boost dose** | **Visit 5** | **Visit 6** | **End of study** |
| **Day ± window** | 0 to 7 days before boost vaccination | 0 | 7±2 | 14 ±3 | 28±3 | 56±10 | 180±10 | 360±14 |
| **Screening for eligibility** |  |  |  |  |  |  |  |  |
| Eligibility check | X |  |  |  |  |  |  |  |
| Informed consent | X |  |  |  |  |  |  |  |
| PCR |  | X |  |  | X |  |  |  |
| **Baseline procedures** |  |  |  |  |  |  |  |  |
| Randomization |  | X |  |  |  |  |  |  |
| Record demographic information | X |  |  |  |  |  |  |  |
| Record medical history | X |  |  |  |  |  |  |  |
| Record medication history | X | X | | | | | | X |
| Complete physical examination | X |  |  |  | X |  |  |  |
| Pregnancy test in urine | X |  |  |  | X |  |  |  |
| Measure vital signs^1^ | X | X | | | | | | X |
| Lab determinations | X^2^ | X^2^ |  |  | X | X |  |  |
| Vaccination |  |  |  |  | X |  |  |  |
| **Endpoint assessment** |  |  |  |  |  |  |  |  |
| Assess acute reactions for at least 15 minutes after study intervention administration |  |  |  |  | X |  |  |  |
| Solicited local and systemic adverse events (AEs) for 7 days after vaccine. Via E-diary |  |  |  |  | X (7 days) |  |  |  |
| Unsolicited local and systemic adverse events (AEs) for 28 days after vaccine . Via E-diary |  |  |  |  | X^3^ | |  |  |
| Unsolicited SAEs |  | X | | | | | | |
| Medically-attended adverse events (MAAEs) |  | X | | | | | |  |
| Immune response evaluation |  | X | X | X | X | X | X | X |

Note: If subjects in Group 2 are not crossed over, the visits will be the same as the groups 1.

1. Temperature, blood pressure and heart rate

2. Lab determination could be performed either at screening vist or randomization

3. Two days per week

**Participants with COVID19-like signs and symptoms**

| **Procedure** | **Symptoms** | **Confirmation** | **Early Follow up** | **Late Follow up** |
| --- | --- | --- | --- | --- |
| **Day ± window** | Day 1-2 | Day 2-5 | Day 6 to 28 | Day 29±7 |
|  |  |  |  |  |
| Site to contact participant if COVID19 signs or symptoms are recorded in the e-Diary | X |  |  |  |
| Case evaluation | X |  |  |  |
| Nasal swab sample |  | X |  |  |
| Humoral immunity |  | X |  | X |
| Biomarker RNAseq |  | X |  | X |
| Vital signs, including pulse oximetry |  | X |  | X |
| Symptoms of infection, including highest body temperature over the last 24 hours measured by participant |  | X | | X |

## Trial schematic


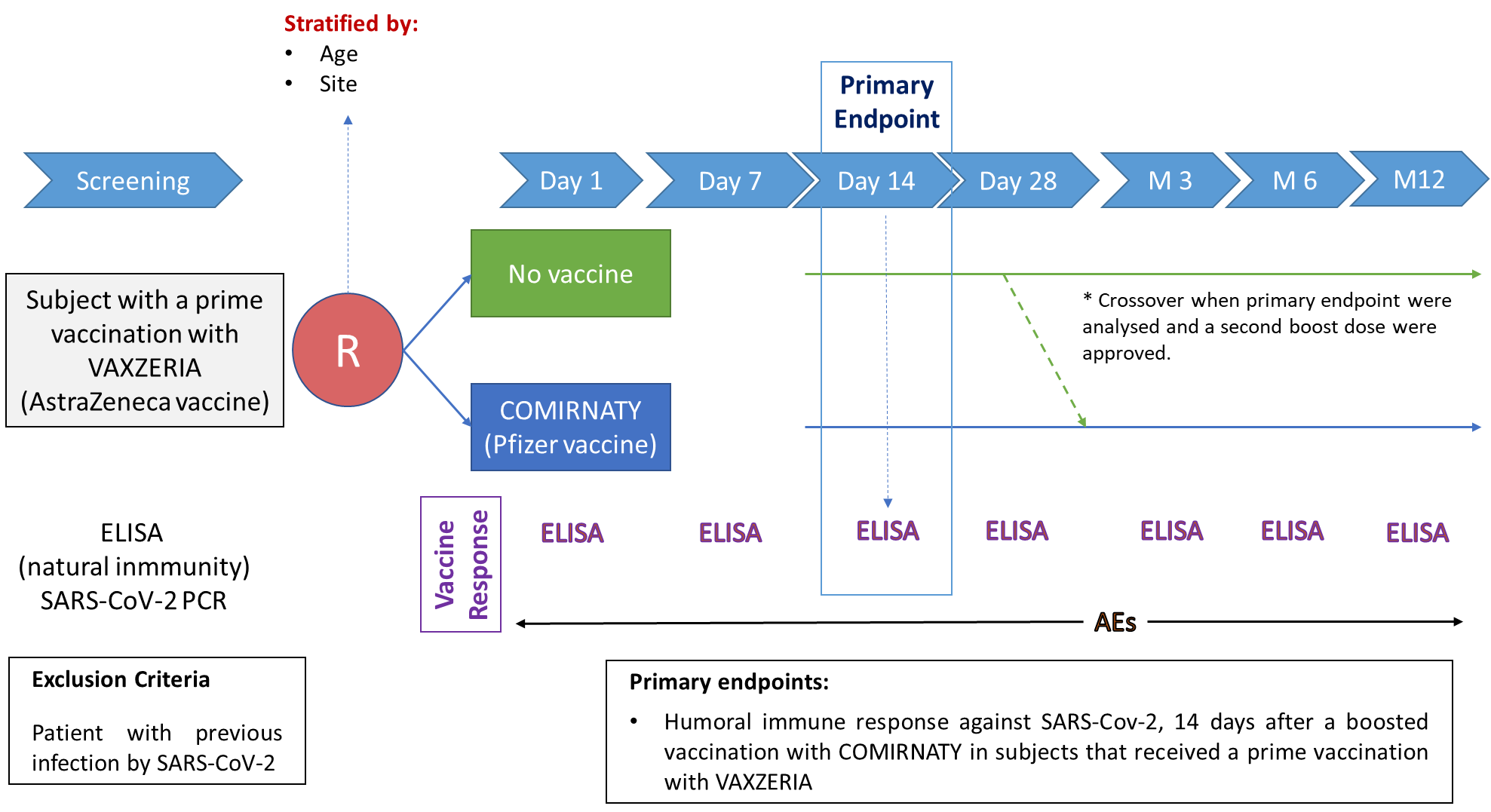


- Sex

Safety

# Introduction

## Study Rationale and Background

Severe acute respiratory syndrome coronavirus 2 (SARS-CoV-2) is the causal agent of coronavirus disease 2019 (COVID-19) outbreak. Declared as a pandemic on March 11, 2019 by the World Health Organisation, COVID-19 outbreak is an evolving global health emergency. A huge effort for prevention and treatment of COVID-19 has since been devoted globally. As of February 16, 2021, almost 5000 trials related with COVID-19 have been registered.

Vaccination against COVID-19 started on 27 December 2020 across the European Union. Four vaccines have been so far given conditional marketing authorisation by the European Commission: mRNA vaccine BNT162b2 (BioNTech and Pfizer - COMIRNATY), mRNA vaccine CX-024414 (Moderna), adenovirus vaccine ChAdOx1-S (AstraZeneca - VAXZEVRIA) and adenovirus vaccine Ad26.Cov2.S (Janssen-Cilag International NV). These vaccines are indicated for active immunisation to prevent COVID-19 caused by SARS-CoV-2 in individuals 16 or 18 years of age and older. Most coronavirus vaccines are given as two injections: an initial ‘prime’ dose followed by a ‘second’ to stimulate the immune system’s memory cells and amplify the immune response (only Ad26.Cov2.S vaccine has been approved as a single-dose). However, their safety and effectiveness using them as heterologous vaccination regimens have not yet evaluated.

Due this pandemic situation, the ability to mix and match vaccines could make vaccination programmes more flexible, speeding up the process and reducing the impact of any supply-chain disruptions. This concept has been also used in other vaccines, as Ebola one. A heterologous prime-vaccination combination of Ebola Virus Vaccine was evaluated in healthy adult subjects in a clinical trial, concluding that the standard and accelerated heterologous prime-boost regimens were well-tolerated and elicited potent cellular and humoral immunogenicity. Therefore, this heterologous prime-boost regimen was approved last year by European regulators to protect against Ebola.

Also, induction of immune response by combining vaccines using different platforms could be stronger. It has been shown that some vaccines produce a better antibodies response while others best stimulate cellular response.

In the case of vaccine against SARS-CoV-2 infection, some trials are ongoing using heterologous prime boost regimen for the prevention of COVID19: 1) NCT04760730 trial use both adenovirus vector vaccines (VAXZEVRIA and rAd26-S), 2) NCT04776317 trial use Chimpanzee Adenovirus serotype 68 (ChAd) and self-amplifying mRNA (SAM) vectors in healthy adult subjects including older adult subjects, and 3) NCT04684446 trial evaluate the safety and immunogenicity of VAXZEVRIA given in combination with (either before or after) rAd26-S.

CombiVacS is the first clinical trial evaluating the immunogenicity and safety of second vaccination with a mRNA COVID19 vaccine (COMIRNATY – Pfizer) in subject having received prime vaccination with a viral vector COVID19 vaccine (VAXZEVRIA – AstraZeneca).

## Benefit/Risk Assessment

While the efficacy and safety profile of the approved COVID-19 vaccines are subject to investigation when used as heterologous vaccination regimen within this protocol, the only contraindication to COVID-19 vaccines has been determined as hypersensitivity to the active substance or to any of the excipients within the vaccine product. Therefore, participants volunteering for this trial are not considered to be at additional risk related to the administration of the COVID-19 vaccine.

Participants in this study are exposed to some general risks. The participants may be subjected to additional blood sampling without representing any relevant harm or discomfort. Collection of other samples, like oropharyngeal or nasal swabs, or urine, are considered not to cause any significant harm.

Participants’ identities will be protected, and personal health information (PHI) held securely. No directly identifiable data will be stored in the clinical trial database, and the participant lists will be stored separately, secured, at the local study sites. The risk that unauthorized persons will see the participant’s PHI will be kept at a minimum, and measures will be taken to keep PHI confidential and in accordance with the legal requirements.

# Objectives and Endpoints

The hypothesis of this trial is that in patients having received a prime dose with VAXZEVRIA immunogenicity after a boosted dose with COMINARTY is better than if no boosted vaccination is done.

**Table 3.1: Objectives and corresponding endpoints**

| **Objectives** | Endpoints |
| --- | --- |
| **Primary Immunogenicity Objective** | |
| To assess the humoral immune response against SARS-CoV-2, 14 days after a vaccination with COMIRNATY in subjects that received a previous single dose of VAXZEVRIA, as compared with no dosing. | - Serological response to vaccination (antibodies against SARS-CoV-2 spike protein) as measured by immunoassay, 14 days after the boost dose. |
| **Exploratory objective** | |
| To evaluate the relationship between the immune response measured as NAV (Neutralizing antibodies) and antibodies against SARS-CoV-2 spike protein measured by immunoassay. | - NAV (Neutralizing antibodies) and immunoassay (enzyme-linked immunosorbent assay). |
| To assess the cellular immune response against SARS-CoV-2, 14 and 28 days after a vaccination with COMIRNATY in subjects that received a previous single dose of VAXZEVRIA, as compared with no dosing. | - Cellular response to vaccination (inflammatory cytokine production IFNg and IL2 against SARS-CoV-2 spike protein) as measured by ELISA immunoassay, basal, 14 and 28 days after the boost dose. |
| **Secondary immunogenicity objectives** | |
| To assess the humoral immune response against SARS-CoV-2, 28 days after a vaccination with COMIRNATY in subjects that received a previous single dose of VAXZEVRIA. | - Serological response to vaccination (antibodies against SARS-CoV-2 spike protein) as measured by immunoassay, 28 days after the boost dose.  - SARS-CoV-2 neutralization as measured by virus neutralization assay, 14 and 28 days after the boost dose. |
| To assess the long-term (up to 1 year) humoral immune response against SARS-CoV-2 of a dose of COMIRNATY in subjects that received a previous single dose of VAXZEVRIA. | -Serological response to vaccination (antibodies against SARS-CoV-2 spike protein) as measured by immunoassay at 3, 6 and 12months after the boost dose.  - SARS-CoV-2 neutralization as measured by virus neutralization assay at 3, 6 and 12 months after the boost dose. |
| To assess the humoral immune response against viral variants of SARS-CoV-2, 14 and 28 days after a dose of COMIRNATY in subjects that received a previous single dose of VAXZEVRIA. | -Serological response to vaccination (antibodies against SARS-CoV-2 spike protein) as measured by immunoassay at 3, 6 and 12months after the boost dose.  - SARS-CoV-2 neutralization as measured by virus neutralization assay at 3, 6 and 12 months after the boost dose. |
| **Secondary efficacy objective** | |
| To assess the occurrence of symptomatic molecularly confirmed COVID-19 and severity of COVID-19 signs and symptoms after the administration of a dose of COMIRNATY in subjects that received a prior single dose of VAXZEVRIA. | - The number of participants with molecularly confirmed COVID-19.  - Presence and severity of COVID-19 signs and symptoms as measured by Symptoms of Infection with Coronavirus-19 (SIC). |
| **Safety** | |
| To evaluate the safety of a dose of COMIRNATY in subjects that received a previous single dose of VAXZEVRIA. | - Solicited local and systemic adverse events (AEs) for 7 days after vaccine  - Unsolicited local and systemic adverse events (AEs) for 28 days after vaccine (Two days per week)  - Serious adverse events (SAEs) throughout the study (from randomization until end of the study).  - Medically-attended adverse events (MAAEs) from the day of vaccination until 6 months after the last vaccination  - Lab parameters: biochemistry and haematology at 1 month visit. |

# Study Design

## Overall Design

This is a randomized, non-blinded, controlled, adaptive, multicenter, Phase II study in subjects aged more than 18 years and in good health or stable clinical situation that have received a previous single dose of VAXZEVRIA.

This is a phase 2 adaptive trial developed for evaluating the immunogenicity of a dose of COMIRNATY after a previous single dose with VAXZEVRIA. A stratification will be made for the following factors: site of vaccination, sex and age. This protocol allows to test the immunogenicity and safety of a heterologous vaccination strategy after a single dose of VAXZEVRIA.

Subjects will be randomized to immediately receive or not dose of COMIRNATY in a proportion of 2:1.

If primary analysis at 14 days confirms the starting hypothesis, subjects randomized to no vaccination will be considered for administration of one dose of COMINARTY at day 28th according to Public Health Department of the Ministry of Health recommendations on heterologous vaccination. If vaccinated, subjects will be followed at the time points defined in the flowchart.

In case the primary analysis does not confirm the starting hypothesis, subject will be followed without administration of COMINARTY.

In case Public Health Department gave instructions to give the second dose of VAXZEVRIA to those subjects having received the first dose, subject will be followed as indicated in flowchart of Group 2.

Other heterologous vaccination strategies could be incorporated if deemed necessary for public health reasons. This could include the use of different boost strategies including those already marketed vaccines for comparative assessment of their safety and efficacy on SARS-CoV-2 and its variants.

The different intervention arms planned (and its corresponding control arm) can be carried-out in different trial sites, thus not all trial sites must participate in all arms.

**Selection sample:**

A multistage cluster sampling stratified by age groups will be carried out to obtain the number of subjects to be selected from the list of 400,000 subjects registered by Spanish Government as having received only the first dose of AZ vaccine. The first stage will be the autonomous community, the second the postcode and the third the hospital centre. Subjects will be selected in this last stage by systematic sampling stratified by age group (18-49, 50-59, and 60 and over years).

**Randomisation and blinding:**

- Randomization will be centralized, and each participant will be randomized stratified by age and centre. Randomization will be in a proportion of 2:1 (COMIRNATY:No vaccination)
- This is an open-label trial.

## Scientific Rationale for Study Design

There is an urgent need to take decisions about how to proceed in those subjects having received a first dose of VAXZEVRIA during the Covid-19 vaccination campaign in Spain, after the decision taken by public health authorities put on hold the second dose of Vaxzevria. In addition, the ability to mix and match vaccines could make vaccination programmes more flexible, speeding up the process and reducing the impact of any supply-chain disruptions. At this moment no information exists on the safety and immunogenicity response for heterologous vaccination schemes. Therefore, a pragmatic trial is proposed to obtain enough information for public rapid decision making and to allow long-term evaluation on the safety and immunogenicity response after the boost dose of a mRNA vaccine (COMINARTY) in these subjects.

## Justification for Dose

The dose of COMINARTY to be used for vaccination is the approved dose of this vaccine.

## End of Study Definition

**Participant Completion**

A participant is considered to have completed the study if he/she has completed all study 1 year visits.

Study completion.

The study is considered complete when the last participant entering the trial has completed his/her last visit.

# Study Population

Adult subjects (18 years old or higher) in good health or stable clinical situation volunteering to participate in the trial will be recruited after giving informed consent.

Selection criteria for inclusion of subjects in the trial will be maintained as pragmatic as possible.

## Inclusion Criteria

Each potential participant must satisfy all of the following criteria to be enrolled in the study:

1. Adult subjects (18 years old) having received a prime VAXZEVRIA vaccination between 8 and 12 weeks before the screening visit.
2. Participants must provide consent indicating that he or she understands the purpose, procedures and potential risks and benefits of the study, and is willing to participate in the study.
3. Subjects in good health or stable clinical situation.
4. Participant is willing and able to adhere to the procedures specified in this protocol.

## Exclusion criteria

Any potential participant who meets any of the following criteria will be excluded from participating in the study:

1. Participant has a clinically significant acute illness (this does not include minor illnesses such as diarrhea or mild upper respiratory tract infection) or temperature ≥38.0ºC within 24 hours prior to the planned dose of study vaccine.
2. Participant has a known or suspected allergy or history of anaphylaxis or other serious adverse reactions to COMIRNATY excipients.
3. Subjects with any contraindication to the administration of COMIRNATY, included pregnancy.
4. Subjects with prior documented COVID19 since VAXZEVRIA vaccination.
5. Subjects have symptoms or signs compatible with COVID19.
6. Subjects participating in a clinical trial in the last three months.
7. Any condition or situation precluding or interfering the compliance with the protocol.

# Study Interventions and Concomitant Therapy

Study intervention is defined as heterologous boosted strategy based on approved SARS-CoV-2 vaccine intended to be administered to a study participant according to this protocol.

## Study Intervention(s) Administered

### Intervention arms:

Intervention arms (Group 1) will be the arm vaccinated with COMIRNATY after a previous vaccination with VAXZEVRIA between 8 and 12 weeks before the screening visit.

### Control arm:

The control arm (Group 2) will have no vaccine administration. I If primary analysis at 14 days confirms the starting hypothesis, subjects randomized to no vaccination will be considered for administration of one dose of COMINARTY at day 28^th^ according to Public Health Department of the Ministry of Health recommendations on heterologous vaccination. If vaccinated, subjects will be followed at the time points defined in the flowchart (Group 2).

## Preparation, Handling, Storage, and Accountability

All study vaccine must be stored in a secured location with no access for unauthorized personnel and at controlled temperatures as indicated on the commercial labels and manufacturer instruction. If study vaccine is exposed to temperatures outside the specified temperature range, all relevant data will be sent to the manufacturer and sponsor to determine if the affected supplies can be used or will be replaced. The affected study vaccine must be quarantined and not used until further instruction from the sponsor is received.

Instructions for the preparation, handling and storage will be those described in the Product Information Sheet of COMIRNATY approved by AEMPS.

The investigator is responsible for ensuring that all study vaccine received at the site is inventoried and accounted for throughout the study. The study vaccine administered to the participant must be documented on the vaccine accountability form. All study vaccine will be stored and disposed of according to the manufacturer’s instructions.

## Measures to Minimize Bias: Randomization

This is an open-label clinical trial.

The randomization list will be generated using software SAS 9 for Windows. The randomization list will be imported into the REDCap program so the researchers can randomize candidate subjects using an easier-to-use interface. Subjects that meet the selection criteria will be randomized in a 2:1 ratio between the two treatment groups (Intervention arm: administration of a COMINARTY boosted dose vs Control arm: no administration of boosted dose), stratifying by center, gender and age (18-49 years, 50-59 years and ≥60 years), in order to achieve balanced randomization in the two treatment groups. The assignment of treatment to each subject will be centralized, keeping the sequence hidden.

## Study Intervention Compliance

This study will be conducted within hospitals. Treatment is administered at site and participants will receive study intervention directly from the investigator or designee, under medical supervision. The date and time of each dose administered in the clinic will be recorded in the source documents. Deviation(s) from the prescribed dosage regimen should be recorded. Intervention start and stop dates, will also be recorded.

## Concomitant Therapy

Any medication or vaccine (including over the counter or prescription medicines and/or recreational drugs) at the time of enrolment or received during the study must be recorded along with:

- Reason for use
- Dates of administration including start and end dates
- Dosage information including dose and frequency

# Discontinuation of Study Intervention and Participant Discontinuation/Withdrawal

## Discontinuation of Study Intervention

This is not applicable. Intervention is a unique dose of COMIRNATY in the randomization visit.

## Participant Discontinuation/Withdrawal from the Study

A participant may withdraw from the study at any time at his/her own request or may be withdrawn at any time at the discretion of the investigator for safety, behavioural, or compliance reasons. This is expected to be uncommon.

When a participant withdraws before study completion, the reason for withdrawal is to be documented in the eCRF and in the source document. If the reason for withdrawal from the study is withdrawal of consent, then no additional assessments are allowed. The participant will be permanently discontinued from the study intervention and the study at that time.

If the participant withdraws consent for disclosure of future information, the sponsor may retain and continue to use any data collected before such a withdrawal of consent.

If a participant withdraws from the study, he/she may request destruction of any samples taken and not tested, and the investigator must document this in the study site records.

## Lost to Follow-Up

To reduce the chances of a participant being deemed lost to follow-up, prior to randomization attempts should be made to obtain contact information from each participant, eg, home, work, and mobile telephone numbers and email addresses for both the participant as well as appropriate family members.

A participant will be considered lost to follow-up if he/she is unreachable for the study site per telephone directly to the patient or relatives (for at least in two occasions), or by written correspondence. A participant cannot be deemed lost to follow-up until all reasonable efforts made by the study-site personnel to contact the participant are deemed futile.

# Study Assessments and Procedures

**General instructions**

Study procedures and their timing are summarized in Flow Chart (section 1.2). Protocol waivers or exemptions are not allowed.

Safety concerns should be discussed with the sponsor or sponsor representative immediately upon notice, to determine if the participant should continue or discontinue the study intervention.

Adherence to the study design requirements, including those specified in the flow chart, is essential and required for study conduct.

**Screening visit**

Screening for eligible participants will be performed within ≤7 days before randomization. The inclusion and exclusion criteria for enrolling participants in this study are described in section 5. If there is a question about these criteria, the investigator must consult with the sponsor (instituto de Salud Carlos III) representative and resolve any issues before enrolling a participant in the study. Waivers are not allowed.

The study will be explained to the participant and informed consent will be obtained prior to any study procedure.

After screening and obtaining informed consent, the following procedures will be performed:

- Record demographic information.
- Record medical history.
- Record concomitant medications and vaccinations if taken within 6 months prior to enrolment in this trial.
- Perform a complete physical examination, including height and weight.
- Measure vital signs (body temperature, pulse, blood pressure).
- Pregnancy test

**Visit 1. Randomization**

- PCR
- Review medical history
- Review medication history
- Measure vital signs (body temperature, pulse, blood pressure).
- Blood extraction for immune response evaluation.
- Blood extraction for lab safety determinations.
- Randomization.
- Pregnancy test

**Vaccination arm**

- Review criteria for cancellation of vaccination.
- Administer the trial vaccine dose according to the subject’s assignment and administration guidance.
- Observe the subject on site for at least 15 minutes following vaccination for safety monitoring. At the end of the observation period, record the occurrence of any AEs following trial vaccination.

**Visit 2 (Day 7±2)**

- Inquire about AEs and general well-being of the participant since the administration of vaccine dose. Record any AEs, ‘solicited’ and ‘unsolicited’ AEs, SAEs and MAAE.
- Review medication history
- Measure vital signs (body temperature, pulse, blood pressure).
- Blood extraction for immune response evaluation

**Visit 3 (Day 14±3)**

- Inquire about AEs and general well-being of the participant since the administration of vaccine dose. Record any AEs, ‘unsolicited’ AEs, SAEs and MAAE.
- Review medication history
- Measure vital signs (body temperature, pulse, blood pressure).
- Blood extraction for immune response evaluation

**Visit 4 (Day 28±3)**

- Inquire about AEs and general well-being of the participant since the administration of vaccine dose. Record any AEs, ‘unsolicited’ AEs, SAEs and MAAE.
- Review medication history
- Measure vital signs (body temperature, pulse, blood pressure).
- Blood extraction for immune response evaluation
- Lab determination

**Control arm (Group 2)**

- Review criteria for cancellation of vaccination.
- PCR
- Administer the trial vaccine dose according to the subject’s assignment and administration guidance.
- Observe the subject on site for at least 15 minutes following vaccination for safety monitoring. At the end of the observation period, record the occurrence of any AEs following trial vaccination.

**Visit 5 (Day 90±10 or 56±3):**

- Inquire about AEs and general well-being of the participant since the administration of vaccine dose. Record any AEs, ‘solicited’ and ‘unsolicited’ AEs, SAEs and MAAE.
- Review medication history
- Measure vital signs (body temperature, pulse, blood pressure).
- Blood extraction for immune response evaluation
- Lab determination at 56±3 for group 2 in case of COMIRNATY vaccination

**Visit 6 (Day 180±10):**

- Inquire about AEs and general well-being of the participant since the administration of vaccine dose. Record any AEs, ‘unsolicited’ AEs, SAEs and MAAE.
- Review medication history
- Measure vital signs (body temperature, pulse, blood pressure).
- Blood extraction for immune response evaluation

**Visit 7 (Day 360±10):**

- Inquire about AEs and general well-being of the participant since the administration of vaccine dose. Record any AEs, ‘unsolicited’ AEs, SAEs and MAAE.
- Review medication history
- Measure vital signs (body temperature, pulse, blood pressure).
- Blood extraction for immune response evaluation

## Immunogenicity Assessments

Planned time points for all immunogenecity and clinical efficacy assessments are provided in the the Flow-Chart (section 1.2 Flowchart). Details of the efficacy assessments are provided below.

All samples for immunogenicity test will be sent to “Centro Nacional de Microbiología” for centralized analysis. After all planned test in this protocol are finalized, all samples will be stored in the ISCIII during 15 years. Detailed sampling shipping instructions will be sent to participating sites from the laboratory.

### Primary immunogenicity assessment.

The primary immunogenicity assessment will take place 14 days after the boost dose by serological response to vaccination as measured by immunoassay. All determinations will be performed at central level in “Centro Nacional de Microbiología”.

### Secondary immunogenicity assesments.

Secondary immunogenicity assessments will include:

- Serological response to vaccination as measured by immunoassay, 28 days after the COMIRNATY dose.
- Serological response to vaccination as measured by immunoassay at 3, 6 and 12months after the COMIRNATY dose.
- Neutralizing antibody titers at 14 and 28 days and 3, 6 and 12 months after the COMIRNATY dose.

Exploratory assessment includes:

- Cellular response to vaccination (inflammatory cytokine production IFNg and IL2 against SARS-CoV-2 spike protein) as measured by ELISA immunoassay, basal, 14 and 28 days after the boost dose.

## Efficacy assessments

Occurrence of confirmed COVID-19 is defined as the presence of a documented positive SARS-CoV-2 test performed within a healthcare facility. Classification of severity will be based on the highest degree of severity during the observation period.

In general, adults with SARS-CoV-2 infection can be grouped into the following severity of illness categories. However, the criteria for each category may overlap or vary across clinical guidelines and clinical trials, and a patient’s clinical status may change over time.

- Asymptomatic or Presymptomatic Infection: Individuals who test positive for SARS-CoV-2 using a virologic test (i.e., a nucleic acid amplification test or an antigen test) but who have no symptoms that are consistent with COVID-19.
- Mild Illness: Individuals who have any of the various signs and symptoms of COVID-19 (e.g., fever, cough, sore throat, malaise, headache, muscle pain, nausea, vomiting, diarrhea, loss of taste and smell) but who do not have shortness of breath, dyspnea, or abnormal chest imaging.
- Moderate Illness: Individuals who show evidence of lower respiratory disease during clinical assessment or imaging and who have saturation of oxygen (SpO2 ) ≥94% on room air at sea level.
- Severe Illness: individuals hospitalized needing oxygen by NIV or high flow, re-breather mask.
- Critical Illness: Individuals who have respiratory failure, septic shock, and/or multiple organ dysfunction.

## Safety Assessments

The definitions of an AE and an SAE and MAAE can be found in Table 8.4.1.

Planned time points for all safety assessments are provided in the Flow-Chart. Unscheduled clinical laboratory measurements may be obtained at any time during the study to assess any perceived safety issues.

A clinical assessment, including medical history, will be performed on all participants at his/her first visit to establish a baseline. Significant medical history and observations from any physical examination, if performed, will be documented in the eCRF.

Acute reactions within the first 15 minutes will be assessed and documented in the eCRF.

AEs will be reported by the participant (or, when appropriate, by a caregiver, surrogate, or the participant's legally authorized representative).

The investigator and any qualified designees are responsible for detecting, documenting, and recording events that meet the definition of an AE or SAE and remain responsible to pursue and obtain adequate information both to determine the outcome and to assess whether the event meets the criteria for classification as an SAE or caused the participant to discontinue the study intervention.

AEs will be documented in the eCRF for each visit as determined in the Flowchart.

## Adverse Events (AEs), Serious Adverse Events (SAEs), and Other Safety Reporting

The definitions of the EU Directive 2001/20/EC Article 2 based on the principles of GCP and EU guidance ENTR/CT 3 apply to this trial protocol, when required local requirements should also apply.

Table 8.4.1: Definitions of Adverse Events

| **TERM** | **DEFINITION** |
| --- | --- |
| **Adverse Event (AE)** | Any untoward medical occurrence in a patient or clinical trial participant, which does not necessarily have a causal relationship with the research procedures or the investigational medicinal product (IMP). |
| **Adverse Reaction (AR)** | Any untoward and unintended responses to an IMP related to any dose administered. |
| **Serious Adverse Event or Reaction (SAE/SAR)*** | Any AE/AR that, at any dose, results in:   - death; - a life-threatening AE; - hospitalization or prolongation of existing hospitalization; - a persistent or significant disability or incapacity; - a congenital anomaly/birth defect; - an "important medical event" |
| **Suspected Unexpected Serious Adverse Reaction (SUSAR)** | An unexpected adverse reaction is an AR of which the nature, outcome, frequency or severity is not consistent with the applicable Reference Safety Information (RSI): SmPC or Investigator's Brochure (IB). |
| **Medically Attended Adverse Events (MAAEs)** | Adverse events with medically-attended visits that were not routine visits for physical examination or vaccination, such as visits for hospitalization, an emergency room visit, or an otherwise unscheduled visit to or from medical personnel (medical doctor) for any reason |
| **New fact**** | Any safety data that could modify significantly the evaluation of the benefit/risk ratio of the IMP or the clinical trial, likely to affect the safety of participants or that could modify the IMP administration, the trial documentation or the conduct of the trial, or to suspend, interrupt or modify the protocol or similar trials. |

** EXCEPTIONS: the following events are not considered as SAE requiring immediate reporting to the sponsor:*

*-the participant is formally admitted to a hospital for medical reasons with no seriousness criterion and does not require overnight hospitalization*

*-elective or previously scheduled surgery or medical treatment;*

*-hospitalization for social or administrative reasons;*

*-pre-existing diseases or present conditions detected prior to start of study drug administration and which do not worsen.*

***Example: a SAE which could be associated with the trial procedures and which could modify the conduct of the trial, a significant hazard to the subject population such as lack of efficacy of an IMP used for the treatment of a life-threatening disease, recommendations of the data safety monitoring board, if any, where relevant for the safety of subjects.*

### Time period and frequency for collecting and reporting AE and SAE information

- All AEs and SAEs will be collected from the signing of the informed consent form (ICF) until the follow-up visit at the time points specified in the Flowchart.
- All AEs and SAEs that are related to investigational products will be recorded from the time of ICF signing to end of study or early study discontinuation.
- Any clinically relevant event that occurs between signing of the ICF and the first vaccination will be collected and reported as pre-existing medical condition.
- All information on AEs (non-serious and serious) and pregnancies should be recorded on the appropriate eCRF (eCRF completion will be detailed into eCRF user manual).
- All AEs will be monitored until resolution or clinical stabilization.
- SAE information will be transmitted using a SAE form. The form should be reviewed and signed by an investigator and must be transmitted within 24 hours from initiation of SAE.
- All events that meet the definition of a SAE will be reported as SAEs, regardless of whether they are protocol-specific assessments.

### Method of detecting AEs and SAEs

- Care will be taken to not introduce bias when detecting AEs and/or SAEs. Open-ended and non-leading verbal questioning of the participant is the preferred method to inquire about AE occurrences.

### Assessment of AEs and SAEs

**Seriousness**

For any adverse event, the investigator must determine whether the event meets one or more of the seriousness criteria described in Table 8.

Table 8. AE severity scale

| **Grade 1**  **(Mild)** | Mild or transient discomfort, without limitation of normal daily activities; no medical intervention or corrective treatment required. |
| --- | --- |
| **Grade 2**  **(Moderate)** | Mild to moderate limitation of normal daily activities;  minimal medical intervention or corrective treatment required. |
| **Grade 3**  **(Severe)** | Marked limitation of normal daily activities; medical intervention and corrective treatment required, possible hospitalization. |
| **Grade 4**  **(Life-threatening)** | Severe limitation of normal daily activities; medical intervention and corrective treatment required, almost always in a hospital setting. |

**Causality**

The investigator must assess the causality of all AEs/SAEs in relation to IMP, research procedures, and concomitant medications, using the following guidelines:

- **Related** – The AE is known to occur with the study intervention, there is a reasonable possibility that the study intervention caused the AE, or there is a temporal relationship between the study intervention and event. Reasonable possibility means that there is evidence to suggest a causal relationship between the study intervention and the AE.
- **Not Related** – There is not a reasonable possibility that the administration of the study intervention caused the event, there is no temporal relationship between the study intervention and event onset, or an alternate aetiology has been established.

All AEs/SAEs for which the investigator or the sponsor considers a causal relationship to be a reasonable possibility are considered suspected SARs.

Assessment on expectedness is usually done by the sponsor.

## Regulatory reporting requirements for serious adverse events

The sponsor assumes responsibility of reporting AE s and SAEs to the corresponding regulatory authorities. At the same time, the sponsor must report to the investigator all suspected unexpected serious adverse reactions, known as SUSARs. SUSARs must be reported as well to the corresponding IEC/IRB unless otherwise indicated by institution.

# Statistical Considerations

The statistical analysis plan (SAP) will be finalized prior to database lock and it will include a more technical and detailed description of the statistical analyses described in this section. This section is a summary of the planned statistical analyses of the most important endpoints including primary and key secondary endpoints.

## Statistical Hypotheses

The efficacy hipotesis is:

Ho: G(Y1) / G(Y2) = 1 versus H1: G(Y1) / G(Y2) > 1

With definition:

G(Y1) is the titer of antibodies binding to the SARS-CoV-2 S protein at 14th day after a dose of COMIRNATY in subjects that received a prior single dose of VAXZEVRIA.

G(Y2) is the titer of antibodies binding to the SARS-CoV-2 S protein at 14th day in subjects no receiving the COMIRNATY dose at randomization.

The antibodies binding to the SARS-CoV-2 S protein will be measured as lognormal distribution.

## Analysis Sets

For the purposes of analysis, the following analysis sets are defined:

| **Participant Analysis Set** | **Description** |
| --- | --- |
| Full analysis set | - All participants having received at least one dose of study intervention and who have at least one efficacy evaluation after baseline. Participants will be included in the analyses according to the planned randomized intervention. |
| Safety analysis set | - All participants who are exposed to a study intervention. Participants will be analysed according to the intervention they actually received. |

The full analysis set is used to analyse endpoints related to the efficacy objectives and the safety analysis set is used to analyse the endpoints and assessments related to safety.

The following data points sets are defined:

| **Defined Data Points Sets** | **Description** |
| --- | --- |
| DPS1 | - All observed data will be included in the analysis set. |
| DPS2 | - For participants who discontinue study intervention and/or receive rescue therapy, post-discontinuation or post-rescue observations will not be included. |

Full analysis set and DPS1 are used to estimate the primary endpoints and the secondary endpoints for secondary core objective.

Full analysis set and DPS2 are used to for sensitivity analyses.

Safety analysis set and DPS1 are used to present safety data.

## Statistical Analyses

### General considerations

All analyses will be carried out using the statistical software SAS, version 9.4 of the SAS system for Windows.

All continuous variables will be summarized using the following descriptive statistics: n (non-missing sample size), mean, standard deviation (SD), 1st quartile, median, 3rd quartile, maximum and minimum. Absolute and relative frequencies (based on the non-missing sample size) of observed levels will be reported for all categorical variables.

All continuous variables will be tested for normality hypothesis assumption with the kolmogorov-Smirnov test. The antibodies binding to the SARS-CoV-2 S protein will be measured as lognormal distribution to obtain more nearly symmetric distribution.

The number of participants and the flowchart of the study will be presented. The period of enrolment and the total number of participants screened to be included in the study will be presented. The number of ineligible participants and the total number of randomized participants will be presented. The number of participants who never receive the study intervention will also be presented by treatment group and those who were included in the full analysis set and the safety analysis set will be presented.

Baseline is considered as the day 1 visit and the baseline data is data collected just before intervention at day 1. Participants with missing baseline value will be excluded from the analysis.

### Primary endpoint(s) analysis

The immune response measured by immunoassay 14 days after randomisation between the two arms will be tested by one-tailed t-test assuming symmetric distribution of lognormal transformation. Geometric means will be calculated as the mean of the assay results after making the logarithm transformation and then exponentiating the mean to express results on the original scale. One-sided 95% CIs will be obtained by taking natural log transforms of concentrations/titers, calculating the 95% CI with reference to the t-distribution, and then exponentiating the confidence limits. In other case, Mann-Whitney-Wilcoxon test will be used.

### Secondary endpoint(s) analysis

Secondary immunogenicity endpoints (serological response and neutralizing antibodies at each time point) between the two arms will be tested by one-tailed t-test assuming symmetric distribution of lognormal transformation. Geometric means will be calculated as the mean of the assay results after making the logarithm transformation and then exponentiating the mean to express results on the original scale. One-sided 95% CIs will be obtained by taking natural log transforms of concentrations/titers, calculating the 95% CI with reference to the t-distribution, and then exponentiating the confidence limits. In other case, Mann-Whitney-Wilcoxon test will be used.

For secondary efficacy objectives, the absolute and relative frequencies of individuals with molecularly confirmed COVID-19 and SIC will be compared through the two-tailed chi-square test and alpha error equal to 0.05.

### Exploratory/subgroup analysis

No subgroup analysis will be carried out.

### Safety analyses

Safety endpoints include serious adverse events (SAEs) leading to study treatment discontinuation and death. SAEs will be coded using the current version of the Medical Dictionary for Regulatory Activities (MedDRA). SAEs leading to study treatment discontinuation and death will be summarized with absolute and relative frequencies (%) with 95% confidence intervals (CIs). More details on the statistical analysis will be provided in the SAP.

## Interim Analysis

No formal interim analysis for efficacy will be done. However, an independent data monitoring committee consisting of independent scientists not otherwise involved in the study has been appointed and will review the data regularly during the study for safety and scientific integrity and will make recommendations to the sponsor regarding the stopping of an intervention for harm or for futility. The frequency of the committee data review meetings and other aspects such as stopping rules will be detailed in a separate charter depending on the safety profile of the study intervention(s).

## Sample Size Determination

Sample size calculation for a log-transformed outcome measure (Wolfe R, Carlin JB. Sample-size calculation for a log-transformed outcome measure. Control Clin Trials. 1999 Dec;20(6):547-54. doi: 10.1016/s0197-2456(99)00032-x. PMID: 10588295) has been performed to assess the humoral immune response against SARS-CoV-2 14 days after dose of COMIRNATY in subjects that received a prior single dose of VAXZERIA, as compared with no dosing. Sample size to obtain will be 600 participants divided in 400 in arm1 and 200 in arm2 (2:1 randomization ratio): we assume that: a) increase in antibodies titer would be at least x1.35 in subjects receiving the dose of COMIRNATY G(Y1) in relation with those not receiving it G(Y2); b) a coefficient of variation equal to 1.2 or 1.0 and similar between arms, c) a 80% statistical power and alpha equal to 0.01 for one side test (H1: G(Y1)/G(Y2) >1). See table below for different assumptions in coefficient of variation and G(Y1)/ G(Y2). The planned sample size is also considered appropriate to evaluate safety endpoints.

The sample size will be increased by 15% of the participants being non-evaluable.

| **Case** | **alfa** | **beta** | **2:1** | **Coef. Var** | **G(Y1)/G(Y2)** | **VAXZERIA group (n=)** | **COMIRNATY group (n=)** |
| --- | --- | --- | --- | --- | --- | --- | --- |
| **2** | 0.01 | 0.2 | 2 | 1.2 | 1.35 | 173.506 | 347.012 |
| **3** | 0.01 | 0.2 | 2 | 1.2 | 1.40 | 138.026 | 276.052 |
| **4** | 0.01 | 0.2 | 2 | 1.2 | 1.45 | 113.186 | 226.372 |
| **5** | 0.01 | 0.2 | 2 | 1.2 | 1.50 | 95.050 | 190.100 |
| **7** | 0.01 | 0.2 | 2 | 1.1 | 1.35 | 154.248 | 308.496 |
| **8** | 0.01 | 0.2 | 2 | 1.1 | 1.40 | 122.706 | 245.412 |
| **9** | 0.01 | 0.2 | 2 | 1.1 | 1.45 | 100.623 | 201.247 |
| **10** | 0.01 | 0.2 | 2 | 1.1 | 1.50 | 84.500 | 169.000 |
| **12** | 0.01 | 0.2 | 2 | 1.0 | 1.35 | 134.827 | 269.653 |
| **13** | 0.01 | 0.2 | 2 | 1.0 | 1.40 | 107.256 | 214.513 |
| **14** | 0.01 | 0.2 | 2 | 1.0 | 1.45 | 87.954 | 175.908 |
| **15** | 0.01 | 0.2 | 2 | 1.0 | 1.50 | 73.861 | 147.722 |

# Supporting Documentation and Operational Considerations

## Appendix 1: Regulatory, Ethical, and Study Oversight Considerations

### Regulatory and ethical considerations

- This study will be conducted in accordance with the protocol and with the following:
  - Consensus ethical principles derived from international guidelines including the Declaration of Helsinki and Council for International Organizations of Medical Sciences (CIOMS) international ethical guidelines
  - Applicable ICH Good Clinical Practice (GCP) guidelines
  - Applicable laws and regulations

The protocol, protocol amendments, ICF, investigator’s brochure and other relevant documents (eg, advertisements) must be submitted to a Research Ethics Committee (REC) and National Competent Authority (NCA) by the sponsor and reviewed and approved by the REC and NCA before the study is initiated.

Any amendments to the protocol will require REC and NCA approval before implementation of changes made to the study design, except for changes necessary to eliminate an immediate hazard to study participants.

Protocols and any substantial amendments to the protocol will require health authority (NCA) approval prior to initiation except for changes necessary to eliminate an immediate hazard to study participants.

The sponsor will be responsible for the following:

- - Providing written summaries of the status of the study to the REC/NCA annually or more frequently in accordance with the requirements, policies, and procedures established by the IRB/IEC
  - Notifying the REC/NCA of SAEs or other significant safety findings as required by REC/NCA procedures
  - Providing oversight of the conduct of the study at the sites and adherence to requirements of ICH guidelines, the REC/NCA, European Directive 2001/20/EC and European Regulation 536/2014 for clinical trials on medicinal products, and all other applicable local regulations

### Financial Disclosure

Investigators and sub-investigators will provide the sponsor with sufficient, accurate financial information as requested to allow the sponsor to submit complete and accurate financial certification or disclosure statements to the appropriate regulatory authorities. Investigators are responsible for providing information on financial interests during the course of the study and for 1 year after completion of the study.

### Informed Consent process

The investigator or his/her representative will explain the nature of the study, including the risks and benefits, to the participant or their legally authorized representative (LAR) and answer all questions regarding the study.

Participants must be informed that their participation is voluntary. Participants, their LAR or their designated representative (relative) will be required to give a statement of informed consent that meets the requirements, local regulations, ICH guidelines, privacy and data protection requirements, where applicable, and the REC or study centre.

The medical record must include a statement that informed consent was obtained before the participant was enrolled in the study, the nature of consent (participant, LAR, designed representative or independent doctor), and the date the consent was obtained. The authorized person obtaining the informed consent must also sign the ICF. In case of urgent oral consent is obtained it should be written in medical record.

Participants must be reconsented to the most current version of the ICF(s) during their participation in the study if relevant.

A copy of the ICF(s) must be provided to the participant, their legally authorized representative or their designated representative.

Participants who are rescreened are required to give a new consent.

The ICF will contain a separate section that addresses the secondary use of data and remaining samples for optional exploratory research. The investigator or authorized designee will explain to each participant the objectives of the exploratory research. Participants will be told that they are free to refuse to participate and may withdraw their consent at any time and for any reason during the storage period. A separate signature will be required to document a participant’s agreement to allow any data and remaining specimens to be used for secondary exploratory research. Participants who decline to participate in this optional research will not provide this separate signature.

### Data protection

Participants will be assigned a unique identifier by the sponsor. Any participant records or datasets that are transferred to the sponsor will contain the identifier only; participant names or any information which would make the participant identifiable will not be transferred. Sponsor staff that require access to personal data will agree to keep confidentiality. Data relevant to fulfil the objectives of the study will be collected only.

The participant must be informed that his/her personal study-related data will be used by the sponsor in accordance with local data protection law. The level of disclosure must also be explained to the participant who will be required to give consent for their data to be used as described in the informed consent.

The participant must be informed that his/her medical records may be examined by Clinical Quality Assurance auditors or other authorized personnel appointed by the sponsor and by inspectors from regulatory authorities. Study participants have the right to request access to their personal data and the right to request rectification of incorrect or incomplete data.

Exploratory research data will not be returned to participants or investigators, unless required by law or local regulations. Privacy and confidentiality of data generated in the future on stored samples will be protected by the same standards applicable to all other clinical data.

### Data quality assurance

- All participant data relating to the study will be recorded on electronic CRFs. The investigator is responsible for verifying that data entries are accurate and correct by physically or electronically signing the CRF.
- Guidance on completion of eCRFs will be provided in the Trial Master File.
- The investigator must permit study-related monitoring, audits, REC/NCA review, and regulatory agency inspections and provide direct access to source data documents.
- Monitoring details describing strategy, including definition of study critical data items and processes (eg, risk-based initiatives in operations and quality such as risk management and mitigation strategies and analytical risk-based monitoring), methods, responsibilities, and requirements, including handling of noncompliance issues and monitoring techniques (central, remote, or on-site monitoring) are provided in the monitoring plan.
- The sponsor or designee is responsible for the data management of this study, including quality checking of the data.
- The sponsor assumes accountability for actions delegated to other individuals (eg, contract research organizations).
- Records and documents, including signed ICFs, pertaining to the conduct of this study must be retained by the investigator for 15 years after study completion unless local regulations or institutional policies require a longer retention period. No records may be destroyed during the retention period without the written approval of the sponsor. No records may be transferred to another location or party without written notification to the sponsor.

### Source documents

- Source documents provide evidence for the existence of the participant and substantiate the integrity of the data collected. Source documents are filed at the investigator’s site.
- Data entered in the eCRF that are transcribed from source documents must be consistent with the source documents or the discrepancies must be explained. The investigator may need to request previous medical records or transfer records, depending on the study. Also, current medical records must be available.
- Definition of what constitutes source data and its origin can be found in monitoring guidelines.
- The investigator must maintain accurate documentation (source data) that supports the information entered in the eCRF.
- Study monitors will perform ongoing source data verification to confirm that data entered into the eCRF by authorized site personnel are accurate, complete, and verifiable from source documents; that the safety and rights of participants are being protected; and that the study is being conducted in accordance with the currently approved protocol and any other study agreements, ICH GCP, and all applicable regulatory requirements.

### Study and site start and closure

**First Act of Recruitment**

- The study start date is the date on which the clinical study will be open for recruitment of participants.
- The first act of recruitment is the informed consent and will be the study start date.

**Study/Site Termination**

- Study sites will be closed upon study completion. A study site is considered closed when all required documents and study supplies have been collected and a study-site closure visit has been performed.
- The investigator may initiate study-site closure at any time, provided there is reasonable cause and sufficient notice is given in advance of the intended termination.
- Reasons for the early closure of a study site by the sponsor or investigator may include but are not limited to:
  - Failure of the investigator to comply with the protocol, the requirements of the REC/NCA or local health authorities, the sponsor’s procedures, or GCP guidelines
  - Inadequate or no recruitment (evaluated after a reasonable amount of time) of participants by the investigator
  - Total number of participants included earlier than expected
- Reasons for early study termination by the sponsor may include but are not limited to:
  - Discontinuation of further study intervention development
  - Occurrence of AEs unknown to date in respect of their nature, severity and duration
  - Medical or ethical reasons affecting the continued performance of the trial, including
    - external evidence indicating efficacy or harm of any of the study interventions
    - a general lack of eligible patients due to vaccination or other reasons

If the study is prematurely terminated or suspended, the sponsor shall promptly inform the investigators, the RECs, the regulatory authorities (NCA), and any contract research organization(s) used in the study of the reason for termination or suspension, as specified by the applicable regulatory requirements. The investigator shall promptly inform the participant and should assure appropriate participant therapy and/or follow-up.

### Publication policy

The study will be registered in a public register (clinicaltrials.gov), in which trial results will be posted. The results of this study will be published in a suitable publication irrespective of findings. Results will also be presented at scientific meetings.

The sponsor will comply with the requirements for publication of study results. In accordance with standard editorial and ethical practice, the sponsor will generally support publication of multicentre studies only in their entirety and not as individual site data. In this case, a coordinating investigator will be designated by mutual agreement.

Authorship will be determined by mutual agreement and in line with International Committee of Medical Journal Editors authorship requirements. Conflicts of interests will be disclosed. The contribution of ECRIN and its national partners will be fairly described in the acknowledgement section or as co-author, depending on the contribution in the trial design, planning and publication.

In line with the EU data sharing policy, individual patient-level data will be shared with the scientific community (either as anonymised or pseudonymised data sets), while maintaining the integrity and privacy of the trial participants and in compliance with the EU General Data Protection Regulation and local rules. Anonymized data will be made available using a data repository, including any programming code used to produce the trial results. Pseudonymized data will be made available in a secure environment, through a controlled access, upon request and evaluation of the requestee’s ability and willingness to maintain the integrity and privacy of the trial participants. Further details of data sharing will be given in a separate data sharing plan.

## Appendix 2: Clinical Laboratory Tests

### Clinical Laboratory Tests

The tests detailed in table 10.2.1 will be performed by the local laboratory.

Additional tests may be performed at any time during the study as determined necessary by the investigator or required by local regulations.

Table 10.2.1: Protocol-required Laboratory Tests

| **Laboratory Tests** | **Parameters** |
| --- | --- |
| Haematology |  |
|  | Haemoglobin (g/dL) |
|  | Platelet count (x 10^9^/L). |
|  | White blood cell count (x 10^9^/L) |
|  | Neutrophils (x 10^9^/L) |
|  | Lymphocytes (x 10^9^/L) and its fractions. |
| Clinical chemistry |  |
|  | K, Potassium (mmol/L) |
|  | Na, Sodium (mmol/L) |
|  | Creatinine (µmol/L) |
|  | Total bilirubin (µmol/L) |
|  | Haemostasis study (at least INR) |
|  | AST, Aspartate aminotransferase or  SGOT, serum glutamic-oxaloacetic transaminase (U/L) |
|  | ALT, Alanine aminotransferase or  SGPT, Serum glutamic-pyruvic transaminase (U/L) |
|  | Glucose (mmol/L) |
| Pregnancy testing |  |
|  | Pregnancy rapid test in urine |

## Appendix 3: Solicited AEs (applicable for injectable IMPs)

| Type | Event | |
| --- | --- | --- |
| Local AEs  (injection or infusion site) | Discomfort  Itching  Redness  Swelling (soft)  Induration (hard)  Blisters | |
| Systemic Clinical AEs following injections/infusions | Temperature  Chills/night sweats  Myalgia/flu-like general muscle aches  Arthralgia  Malaise (excess fatigue)  Headache  Nausea  Vomiting  Nasopharyngitis/Upper respiratory tract infection/cough/sinusitis  Generalised rash  Generalised itching | |
| Systemic Laboratory AEs | Creatinine  ALT  Alkaline phosphatase  Total bilirubin  INR  Glucose  Sodium  Chloride | Haemoglobin  Total White Cell Count  Neutrophils  Lymphocytes  Platelets |

## Appendix 4: Abbreviations

| AE | adverse event |
| --- | --- |
| ALT | alanine aminotransferase |
| AR | Adverse Reaction |
| AST | aspartate aminotransferase |
| CIOMS | Council for International Organizations of Medical Sciences |
| COVID-19 | coronavirus-induced disease first described in 2019 |
| CRF | case report file |
| DPS | Data Point Set |
| DSMC | Data Safety Monitoring Committee |
| DSUR | Development Safety Update Report |
| e.g. | example given |
| eCRF | electronic case report file |
| EMA | European Medicines Agency |
| GCP | Good Clinical Practice |
| IB | Investigator Brochure |
| ICF | informed consent form |
| ICH | International Council for Harmonization |
| IEC | Independent Ethics Committee |
| IMP | Investigational Medicinal Product |
| INR | international normalized ratio |
| IRB | Institutional Review Board |
| K | potassium |
| LAR | Legally Authorized Representative |
| MedDRA | Medical Dictionary for Regulatory Activities |
| Na | sodium |
| NIV | non-invasive ventilation |
| O2 | oxygen |
| PHI | personal health information |
| PI | principal investigator |
| pO2 | partial pressure of oxygen |
| RNA | ribonucleic acid |
| RSI | Reference Safety Information |
| SAE | serious adverse event |
| SAP | statistical analysis plan |
| SAR | serious adverse reaction |
| SARS-CoV-2 | SARS-coronavirus-2 |
| SGOT | serum glutamic-oxaloacetic transaminase |
| SGPT | serum glutamic-pyruvic transaminase |
| SmPC | Summary of Product Characteristics |
| SpO2 | oxygen saturation |
| SUSAR | suspected unexpected serious adverse reaction |
| TOC | table of contents |
| TSG | Trial Safety Group |

## Appendix 5: Protocol Amendment History

The Protocol Amendment Summary of Changes Table for the current amendment is located directly before the table of contents (TOC).

Following amendments will be registered as indicated below:

**Overall Rationale for the Amendment**

Amendment 1: April 15, 2021

Protocol Version: 1.1

This amendment is considered to be nonsubstantial based on the criteria set forth in Article 10(a) of Directive 2001/20/EC of the European Parliament and the Council of the European Union.

| Section # and Name | Description of Change | Brief Rationale |
| --- | --- | --- |
| 1.2 Flowchart | Procedures harmonization | Minor errors in the procedures of flowchart section |
| Apendix 2. Clinical Laboratory Test | Clarification of laboratory test |  |

Amendment 2: May 21, 2021

Protocol Version: 1.2

This amendment is considered to be nonsubstantial

| Section # and Name | Description of Change | Brief Rationale |
| --- | --- | --- |
| Table 3.1: Objectives and corresponding endpoints | Clarification of exploratory objectives | Inclusion of the definition of cellular immunogenicity |
| All the document | Correction of some typographic mistakes |  |
